# The Evolution of Reverse Gyrase Suggests a Nonhyperthermophilic Last Universal Common Ancestor

**DOI:** 10.1093/molbev/msz180

**Published:** 2019-09-03

**Authors:** Ryan J Catchpole, Patrick Forterre

**Affiliations:** 1 Département de Microbiologie, Institut Pasteur, Unité de Biologie Moléculaire du Gène chez les Extrêmophiles (BMGE), Paris, France; 2 Institute for Integrative Biology of the Cell (I2BC), CEA, CNRS, University of Paris-Sud, University of Paris-Saclay, Gif-sur-Yvette, France

**Keywords:** LUCA, hyperthermophiles, evolution, phylogeny, reverse gyrase

## Abstract

Reverse gyrase (RG) is the only protein found ubiquitously in hyperthermophilic organisms, but absent from mesophiles. As such, its simple presence or absence allows us to deduce information about the optimal growth temperature of long-extinct organisms, even as far as the last universal common ancestor of extant life (LUCA). The growth environment and gene content of the LUCA has long been a source of debate in which RG often features. In an attempt to settle this debate, we carried out an exhaustive search for RG proteins, generating the largest RG data set to date. Comprising 376 sequences, our data set allows for phylogenetic reconstructions of RG with unprecedented size and detail. These RG phylogenies are strikingly different from those of universal proteins inferred to be present in the LUCA, even when using the same set of species. Unlike such proteins, RG does not form monophyletic archaeal and bacterial clades, suggesting RG emergence after the formation of these domains, and/or significant horizontal gene transfer. Additionally, the branch lengths separating archaeal and bacterial groups are very short, inconsistent with the tempo of evolution from the time of the LUCA. Despite this, phylogenies limited to archaeal RG resolve most archaeal phyla, suggesting predominantly vertical evolution since the time of the last archaeal ancestor. In contrast, bacterial RG indicates emergence after the last bacterial ancestor followed by significant horizontal transfer. Taken together, these results suggest a nonhyperthermophilic LUCA and bacterial ancestor, with hyperthermophily emerging early in the evolution of the archaeal and bacterial domains.

## Introduction

Understanding the nature of the last universal common ancestor of extant life (LUCA) is one of the most difficult, yet important problems in evolutionary biology. If we were able to determine the genes encoded by the LUCA, we could make important conclusions regarding the evolutionary histories of all living organisms, as well as make predictions about the environment in which the LUCA lived. However, deciphering phylogenetic relationships dating back billions of years is a process fraught with difficulty. Not least because continual mutation over such time periods saturates sequences, erasing earlier phylogenetic signals that may exist, but also because mechanisms such as horizontal gene transfer act to introduce phylogenetic conflict between protein histories, further decreasing our ability to resolve such ancient relationships. Hence, the field of early evolutionary biology is one which is prone to disagreements, even when considering similar data sets. A poignant example of such disagreement comes from the phylogenies of reverse gyrase (RG), the only known hyperthermophile-specific protein, ubiquitously encoded by the genomes of hyperthermophilic organisms and absent from mesophiles (Forterre 2002a; Kaufmann 2006; Brochier-Armanet and Forterre 2007; Heine and Chandra 2009). Understanding the evolutionary history of RG is important as the presence or absence of this gene in ancestral genomes (such as the LUCA) would allow us to infer a crude optimal growth temperature for these long-extinct species. The presence of RG appears to be incompatible with mesophily, and conversely, the absence of RG appears incompatible with hyperthermophily. Thus, the presence of a gene encoding RG would infer a hyperthermophilic or thermophilic lifestyle excluding the option of a mesophilic lifestyle, and the absence, a mesophilic or moderately thermophilic growth condition, to the exclusion of hyperthermophily. This predictive ability is a powerful tool in evolutionary biology, where the optimal growth temperature of long-extinct organisms plays an important role in understanding genome evolution (e.g., “thermoreduction” [Forterre 1995]; protein and RNA evolution [Boussau et al. 2008; Groussin and Gouy 2011], etc.). In order to make inferences about the presence or absence of RG in ancestral organisms, it is therefore vital to have a robust phylogeny for RG. However, the limited genetic data for hyperthermophilic organisms have restricted our ability to make such generalizations, and the small body of literature regarding RG evolution seems to flip-flop between the presence and absence of RG in LUCA.

Early analyses for the presence of RG were carried out experimentally by looking for positive supercoiling activity in cell lysates (Collin et al. 1988; Bouthier de la Tour et al. 1990). Though this allowed the identification of new RG-encoding species, these early experiments failed to detect RG in bacteria (and some archaeal species) due to the presence of the antagonistic DNA gyrase (Guipaud et al. 1997). Later, the discovery of RG activity in bacteria (namely in the *Thermotogales*) suggested that RG may be a more ancestral protein than previously thought, potentially evolving before the divergence of the bacterial and archaeal lineages (Bouthier de la Tour et al. 1991). Even at this early stage, the presence/absence of RG in the LUCA became an important factor in unraveling the nature of the ancestral life on earth (Forterre et al. 1995). The first published phylogeny for RG included 13 sequences, and did not resolve the monophyly of the bacterial and archaeal domains (Forterre et al. 2000), suggesting that RG could not have evolved solely by vertical descent in the two domains, casting doubt over its presence in the LUCA. A later analysis *was* able to recover the bacterial and archaeal monophyly using a data set of 32 RG sequences; however, the domain separation was only weakly supported, and the bacterial tree did not reflect a canonical 16S phylogeny leading the authors to hypothesize an Archaea-to-Bacteria transfer for RG (Brochier-Armanet and Forterre 2007). Subsequently, another group was again able to recover the bacterial–archaeal domain separation in a phylogeny of only 15 sequences (Heine and Chandra 2009). Although these monophyly-recovering analyses suggest the direct descent of RG from the LUCA, the data sets used were small and the intradomain tree topologies were not as would be expected from an ancient protein evolving independently in the two domains. The most complete RG phylogeny to date used a data set of 97 sequences, and identified RG as a candidate LUCA protein due to the recovered monophyly of the bacterial and archaeal domains, prompting the authors to conclude that the LUCA was likely a hyperthermophile (Weiss et al. 2016). Unfortunately, the topology of the recovered tree was not analyzed in-depth and upon closer examination it is clear that this phylogeny suffers the same problems observed previously, namely the branch between Archaea and Bacteria was rather short and the clades produced in the analysis are atypical and do not conform to the canonical 16S or universal protein phylogenies (tree reproduced in supplementary fig. 1, Supplementary Material online). Therefore, the conclusion that RG was encoded by the LUCA is supported weakly, at best.

With the quantity of available genetic data growing exponentially, and increasing effort being made to sequence the genomes of archaeal species (many of which are hyperthermophiles), we thought it important to update the phylogeny of RG, and the evolutionary conclusions this can achieve. Using bioinformatics techniques, we reveal 376 RG sequences from 247 organisms across the bacterial and archaeal domains. Phylogenetic reconstruction of these sequences does not resolve the monophyly of the two domains, but rather reveals multiple potential horizontal transfer events. These results suggest RG was not present in the LUCA, but rather evolved after the divergence of the lineages leading to the LBCA and LACA. We therefore conclude that LUCA was a mesophile or moderate thermophile, with hyperthermophily evolving later, possibly before the emergence of the LACA.

## Results

### Collection and Analysis of RG Sequences

**Fig. 1.**
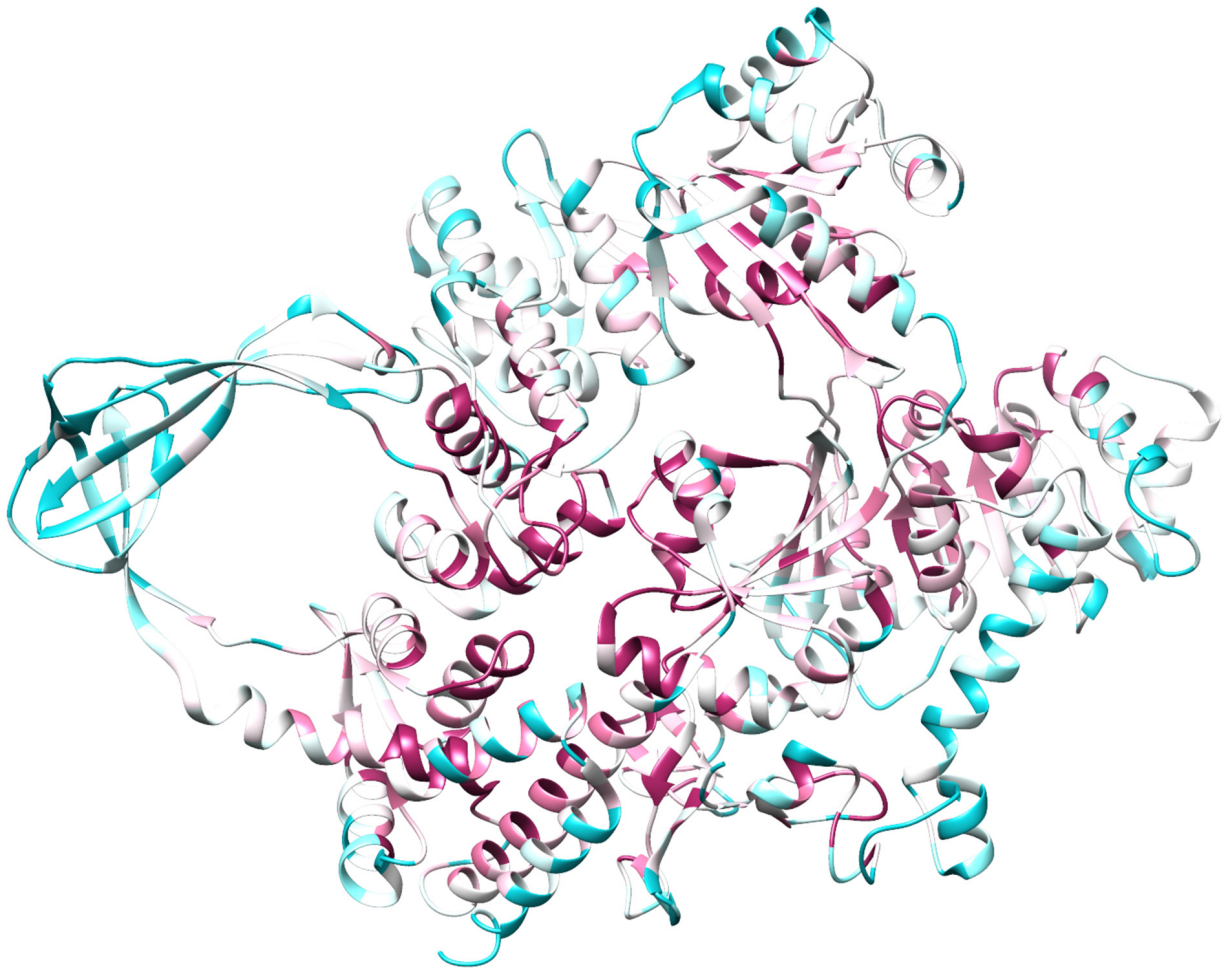
Sequence conservation recovered from alignment of RG data set mapped onto RG structure from *Thermotoga maritima* (pdb: 4DDT). Conserved residues indicated in red shades, less conserved residues indicated in blue shades.

Searching of the nonredundant protein database using a hidden Markov model (HMM) generated with known RG proteins revealed 376 putative RG sequences. These sequences originate from 247 unique species (within-species variants arising from gene duplications and/or differences in start site annotation etc.). Alignment of the amino acid sequences reveals an average pairwise sequence identity of 34.2% across the entire data set, with known helicase and topoisomerase motifs well conserved (supplementary fig. 2, Supplementary Material online). Mapping the degree of sequence conservation observed at each position in our alignment onto the structure of the RG protein from *Thermotoga maritima* pdb: 4DDT (Rudolph et al. 2013) (identical results obtained with RG from the archaeon *Archaeoglobus fulgidus*—pdb: 1GKU; Rodríguez and Stock 2002) reveals a high level of conservation within both the helicase and topoisomerase domains, with less conservation observed at exterior regions (fig. 1). Though this result is not hugely surprising considering the requirement of both domains for RG activity, it does suggest that our data set is likely composed of true RG proteins.

### Species Encoding RG

We were able to obtain information on the optimum growth temperatures of 174 of the 247 species encoding RG. As observed previously, almost all organisms encoding RG are hyperthermophiles or extreme-thermophiles, with 60% of the species in our data set having an optimum growth temperature above 75 °C and 89% above 65 °C. Although difficult to confirm, we believe this data set includes all hyperthermophilic organisms for which genome sequences are available. Thus, our data reaffirm the previous observation that RG is encoded by the genomes of all hyperthermophiles (Forterre 2002a). In addition to extreme-thermophiles, our search also gave hits to RG sequences in five moderate thermophiles with optimum growth temperatures below 65 °C: *Thermodesulfovibrio aggregans* (60 °C; Sekiguchi et al. 2008); *Nitratiruptor tergarcus* (55 °C; Nakagawa et al. 2005); *Lebetimonas natsushimae* (55 °C; Nagata et al. 2017); *Caminibacter mediatlanticus* (55 °C; Voordeckers et al. 2005); *Nautilia profundicola* (40 °C; Smith et al. 2008). The presence of RG in *N. profundicola* has been described previously and is likely an adaptation to short-term exposure to elevated temperatures in hydrothermal vent environments, with RG expression increasing 100-fold during temperature stress at 65 °C (Campbell et al. 2009). As *Ni. tergarcus*, *L. natsushimae*, and *C. mediatlanticus* were also isolated from the walls of active hydrothermal vents, similar adaptive mechanisms may explain the presence of RG in these species.

### Phylogenetic Analysis of RG Data set

**Fig. 2.**
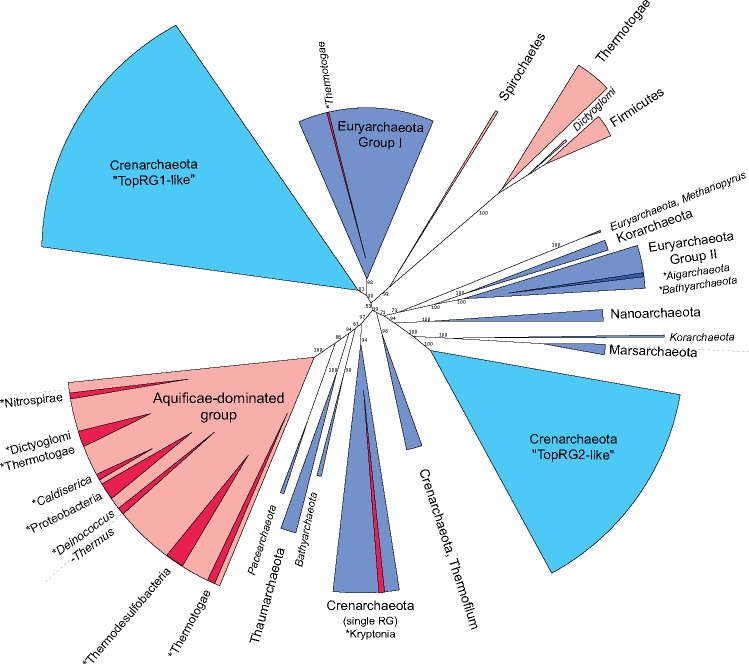
Schematic representation of phylogenetic tree generated using entire RG data set. Archaeal clades colored in blue, Bacterial clades in red, with phyla indicated. Clades formed inside canonical phyla are indicated in darker shades, and labeled with an asterisk. Clades labeled with italicized text indicate ≤2 sequences present. Crenarchaeal TopRG1-like and TopRG2-like paralogues indicated in pale blue. Ultrafast bootstrap values for major bipartitions are indicated on branches. Detailed tree is available in supplementary figure 3 (Supplementary Material online).

**Fig. 3.**
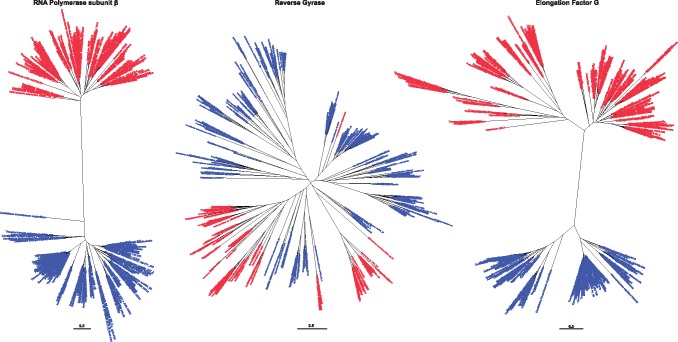
Phylogenetic trees generated using universal proteins from RG-encoding species, compared with RG itself. First panel, RNA Polymerase subunit β; second panel, reverse gyrase; third panel, Elongation Factor G. In all trees, sequences encoded by Archaeal species are indicated in blue, Bacterial species in red.

In order to investigate the evolutionary history of the RG protein, we used our entire data set to generate a single phylogenetic tree. The first feature that is clear to note in our phylogeny is that the bacterial and archaeal sequences are not monophyletic (supplementary fig. 3, Supplementary Material online). The bacterial sequences split into three different clades, as do the archaeal sequences. This is in direct contrast to that expected if RG had emerged in a common ancestor of bacteria and archaea (i.e., the LUCA), and evolved independently since the divergence of these lineages. Although some bipartitions at the center of the tree are not well-supported by ultrafast bootstrap values (supplementary fig. 3, Supplementary Material online), these uncertainties were improved by removing the split RG sequences found in fast-evolving archaea without hugely altering the overall tree topology (fig. 2, supplementary fig. 4, Supplementary Material online) (nor resolving bacterial and archaeal monophyly). To confirm the absence of domain monophyly, we modified the RG tree topology such that Archaea and Bacteria form monophyletic groups, and subjected this tree to various tests of phylogenetic tree selection (unweighted and weighted Kishino–Hasegawa tests [Kishino and Hasegawa 1989]; unweighted and weighted Shimodaira–Hasegawa tests [Shimodaira and Hasegawa 1999]; Expected Likelihood Weight [Strimmer and Rambaut 2002]; and Approximately Unbiased test [Shimodaira 2002]). All tests soundly rejected the monophyletic tree topology (supplementary fig. 5, Supplementary Material online). To further illustrate the contrast between our RG tree and those of proteins inferred to be present in the LUCA, we collected the sequences of two universal marker proteins (rpoB encoding the RNA polymerase β-subunit, and EF-G/aEF-2 encoding translation elongation factor G), as well as the 16S rDNA from the RG-encoding species recovered in our original RG search. In contrast to the RG phylogeny, phylogenetic trees representing these data sets show a clear monophyletic separation of the bacterial and archaeal species (fig. 3, supplementary fig. 6, Supplementary Material online). This is exactly as would be expected for sequences which diverged before the appearance of the LACA and the LBCA (i.e., present in the LUCA). Moreover, this monophyletic tree topology (as obtained with rpoB) is also strongly rejected by all tree selection tests when using the RG sequence data set (even when the interdomain branch length is manually reduced—see below) (supplementary fig. 5, Supplementary Material online).

Perhaps more strikingly than the contrast in monophyly is that the lengths of branches separating the bacterial and archaeal clades are clearly different between our RG phylogeny and those of our universal marker protein phylogenies (fig. 3, supplementary fig. 6, Supplementary Material online). The very long branches displayed by our universal marker proteins are in agreement with the idea that the tempo of evolution was much higher during the period between LUCA and the specific ancestors of Archaea and Bacteria, decreasing later during the diversification of these two domains (Woese 1998; Forterre 2006). This results in the formation of two very divergent versions of universal proteins in Bacteria and Archaea, separated in phylogenetic trees by a very long branch. In contrast, the RG phylogeny has much shorter internode branch lengths, inconsistent with such an evolutionary scenario. This indicates that archaeal and bacterial RG do not form two distinct versions, and likely diverged from each other when the tempo of evolution had already slowed, that is, shortly before or after the formation of the two prokaryotic domains. When combined with the polyphyletic nature of the bacterial and archaeal RG sequences, it becomes clear that RG must have evolved after the time of the LUCA. This is in agreement with that observed in earlier RG phylogenies (Forterre et al. 2000; Brochier-Armanet and Forterre 2007), but contrasts with more recent reconstructions (Heine and Chandra 2009; Weiss et al. 2016).

### Crenarchaeal RG Duplication

An oddity in RG sequences is observed in a large group of Crenarchaeota, including the well-studied Sulfolobales. Here, RG has undergone a gene duplication, and this duplication is clearly represented in our RG phylogenetic tree where the two paralogs form two distinct clades (fig. 2). Additionally, it is apparent from in vitro and in vivo experiments that the two RG paralogs have diverged in function (Bizard et al. 2011; Couturier et al. 2014), with noncomplementary activities. For simplicity, the RG paralogs found in Crenarchaeota are referred to by their nomenclature in *Sulfolobus* species, that is, TopR1-like and TopR2-like. TopR1 has been reported to function as a classical RG, exhibiting ATP-independent topoisomerase activity and DNA renaturation at high temperature; whereas the function of TopR2 is less clear, seemingly exhibiting high levels of ATP-dependent supercoiling at temperatures below those usually required for cell division (Bizard et al. 2011) (with neither being essential in *Sulfolobus islandicus*; Zhang et al. 2018). In order to analyze whether this divergence in activity was mirrored by changes in the RG amino acid sequence, we used data sets limited to each paralog to generate alignments of TopR1-like and TopR2-like proteins. Comparison of these alignments with each other, and with that generated using the complete RG data set reveals that all of the conserved motifs (both helicase and topoisomerase domain motifs; supplementary fig. 2, Supplementary Material online) are present in both TopR1-like and TopR2-like sequences, as well as the active site tyrosine. Despite the similarities, we observed a notable difference in the second putative zinc finger motif of RG (Zn2) of the Crenarchaeal paralogs. The Zn2 motif is conserved in only 62% of our RG sequences, however, it is strictly conserved among all TopR2-like sequences. In contrast, only 35% (23/65) of TopR1-like sequences encode the second cysteine in this CxxCx_9__–__11_CxxC motif, with around half of those (11/23) containing additional inserts within Zn2. This motif has been shown to be important for DNA binding and positive supercoiling, but not for relaxation of negative supercoils or for ATPase activity (Li et al. 2011; Rudolph et al. 2013) and thus may explain the differences in processivity and function of these two enzymes. Due to the apparent functional divergence of TopR1-like and TopR2-like proteins, as well as divergence of TopR2-like proteins from the canonical RG functionality, we thought it important to test the effect of these proteins on the RG phylogeny. Not only could the different evolutionary trajectory of TopR1-like and TopR2-like proteins alter the tree structure (e.g., due to long branch attraction artefacts), TopR2-like proteins do not seem necessary for growth at high temperature (Bizard et al. 2011), and thus may convolute the RG tree due to potentially divergent selective pressures. Removal of these sequences did not resolve the monophyly of the bacterial and archaeal domains, nor did it increase the interdomain branch lengths (supplementary fig. 7, Supplementary Material online). Thus the inclusion of these paralogues has minimal impact on overall tree topology and thus on our conclusions regarding RG presence in LUCA, hence we chose to include these sequences in subsequent analyses.

### Domain-Specific RG Phylogenies

**Fig. 4.**
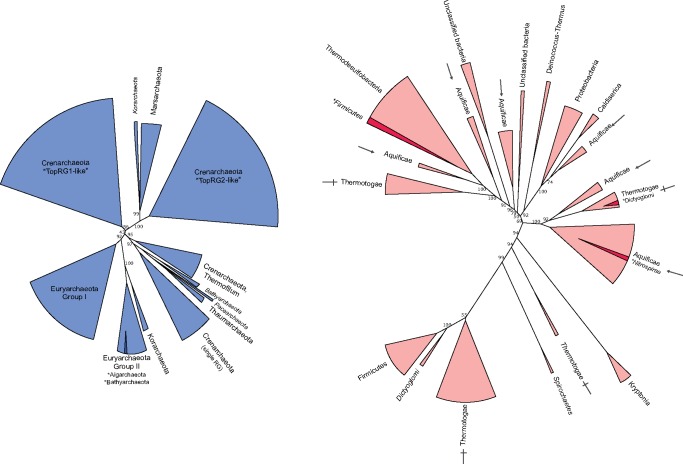
Schematic representation of phylogenetic trees generated using only Archaeal RG sequences (blue), or Bacterial RG sequences (red). Clades formed inside canonical phyla are indicated in darker shades, and labeled with an asterisk. Clades labeled with italicized text indicate ≤2 sequences present. Aquificae and Thermotoga clades are labeled with arrow and cross, respectively, to highlight their paraphyletic nature.

The absence of RG in LUCA suggests that the protein must have emerged in either the lineage leading to the LACA (or a more recent archaeal group), or the lineage leading to the LBCA (or a more recent bacterial group), with horizontal gene transfer (HGT) spreading RG to the second domain. These two scenarios lead to hypotheses testable by further phylogenetic analyses: if RG evolved in the lineage leading to the LACA, an RG phylogeny produced using only archaeal sequences should preserve the canonical archaeal taxonomic groups, whereas the interdomain HGT spreading RG into the bacterial domain would likely not produce a typical bacterial taxonomy (and vice versa). In order to test this hypothesis, we generated RG data sets containing only the 258 archaeal RG sequences, or containing only the 118 bacterial sequences (fig. 4, supplementary figs. 8 and 9, Supplementary Material online).

The archaeal RG phylogeny resulting from this analysis resolves into clades rather congruent with the consensus archaeal phylogeny but with several unexpected positions (supplementary fig. 8, Supplementary Material online). All group I Euryarchaeota (*Thermococcus*, *Methanococcus, Methanobacteriales*) group together, with the exception of *Methanopyrus kandleri* which is known to be fast evolving and difficult to position in the archaeal tree (Gribaldo and Brochier-Armanet 2006) and here groups with three Korarchaea (other fast evolving species). These species cluster with the group II Euryarchaeota, which are positioned between two Crenarchaeotal groups, and contain a RG from a candidatus Bathyarchaeon. Interestingly, removal of the split RG sequences from the archaeal tree restores the expected position of the group II Euryarchaeota (monophyletic with group I Euryarchaeota), perhaps suggesting an artefactual attraction of/by these fast-evolving sequences (supplementary fig. 10, Supplementary Material online). Although removal of split RG sequences recovers a tree topology more congruent with the expected archaeal phylogeny, branch support at some major bipartitions is weakened (supplementary fig. 8 vs. supplementary fig. 10, Supplementary Material online). In both tree topologies, two other bathyarchaeotal RG branch with a Thaumarchaeon and two candidatus *Caldiarchaeum subterraneum*. This grouping is consistent with the monophyly of the BAT (Bathyarchaea, Aigarchaea, Thaumarchaea) clade observed in most phylogenetic analyses (Spang et al. 2015; Da Cunha et al. 2017). However, in the RG phylogeny, this BAT clade branches within Crenarchaeota, reminiscent of the TACK grouping recovered in previous phylogenies (Guy and Ettema 2011). It is unclear if these anomalies are due to some gene transfer events between Archaea (e.g., from Crenarchaea to the BAT group) or from the low resolution of the RG tree at the interphylum level. These results are further difficult to interpret since there is no consensus to the rooting of the archaeal tree (Petitjean et al. 2014; Raymann et al. 2015; Da Cunha et al. 2017; Williams et al. 2017), and thus no “true” phylogeny to which we can compare RG evolution. Regardless, the clear-cut separation between Crenarchaeota and Euryarchaeota (figs. 2 and 4; supplementary fig. 10, Supplementary Material online) is reflective of most canonical archaeal phylogenies and thus might suggest that RG was present in the archaeal domain before the divergence of these phyla. It is unclear from this analysis if RG was already present in the LACA, though the mostly monophyletic nature of the Thaumarchaeota and Bathyarchaeota might suggest this to be the case (though the unrooted nature of this phylogenetic tree does not preclude the possibility of the RG root being present within any of these clades, spreading by horizontal transfer events). More sequences from basal archaeal groups (e.g., Thaumarchaeota and Bathyarchaeota), as well as underrepresented groups (e.g., Nanoarchaeota and Korarchaeota) will help to strengthen the archaeal RG tree, and may allow more unambiguous extrapolation to the LACA.

Notably, the bacterial RG phylogeny follows a much more random pattern of clade separation than the archaeal tree. This is exemplified by the Thermotogales and Aquificales, where members of these orders can be seen in four and six separate clades, respectively (fig. 4, supplementary fig. 9, Supplementary Material online). Moreover, the major clade of Aquificae and that of the Thermodesulfobacteria are separated by quite some distance, with many other clades branching between them. This result is very different from that observed with 16S phylogenies (supplementary fig. 6, Supplementary Material online), and also with our phylogenies based on universal proteins, where these are closely related groups. Furthermore, Thermotoga form a clade together with the Firmicutes—a branching which is not unreasonable; yet the very closely related Pseudothermotoga form a clade within an Aquificae subgroup. It has been observed previously that the phylogenetic positions of Thermotogales and Aquificales can be difficult to ascertain due to the huge influence of horizontal gene transfer during their evolution (Zhaxybayeva et al. 2009). This is reflected in our RG phylogenies, where even closely related species fall within divergent clades suggestive of multiple transfer events sourced from various taxa. Similarly to the Archaea, no universally accepted Bacterial phylogeny is available, and thus comparing the order of bacterial clades resolved with RG is difficult. However, it is clear that the bacterial RG phylogeny does not even conform to canonical taxonomy, and thus is likely highly influenced by gene transfer events.

Taken together, these results suggest that RG has evolved mostly vertically in the Archaea before the divergence of Euryarchaea and Crenarchaea, partly preserving the evolutionary history of the Archaea within its sequence. The evolution of RG in the bacteria has not followed such a pattern of vertical inheritance, rather several horizontal transfer events have resulted in the movement of RG within the bacterial domain.

### Evidence for Horizontal Transfer of RG

The bacterial data set shows further evidence of HGT at the level of individual species or genera. For example, some members of the Aquificae (including *Aquifex aeolicus* and *Hydrogenivirga* sp*. 128-5-R1-1*) encode two copies of RG proteins. In these cases, one of the RG copies is most closely related to other RG proteins encoded by Aquificales; however, the second RG appears most closely related to that of Thermodesulfobacteria (supplementary fig. 3, Supplementary Material online). If these two RG copies had arisen by duplication within an ancestor of *Aquifex* and *Hydrogenivirga*, we might expect the two proteins to be most closely related to each other rather than to RG of other species. Instead, it appears that the second RG copy has arisen by HGT from an ancestor of Thermodesulfobacteria. Alternatively, the two RG sequences could have arisen by duplication within an ancestor of Aquificales, and then each transferred independently to other organisms, thus disrupting the expected tree topology. Either way, it is clear that HGT has played a significant role in the evolution of RG in these groups.

Our RG phylogeny also confirms previously observed evidence for HGT of RG. For example, the interdomain transfer from a Crenarchaeon to an ancestral Kryptonia bacterium (Eloe-Fadrosh et al. 2016), and the noncanonical position of some *Dictyoglomus* species suggesting transfer from a *Fervidobacterium* (Brumm et al. 2016) (supplementary fig. 3, Supplementary Material online).

### Rooting of the RG Phylogenetic Tree

**Fig. 5.**
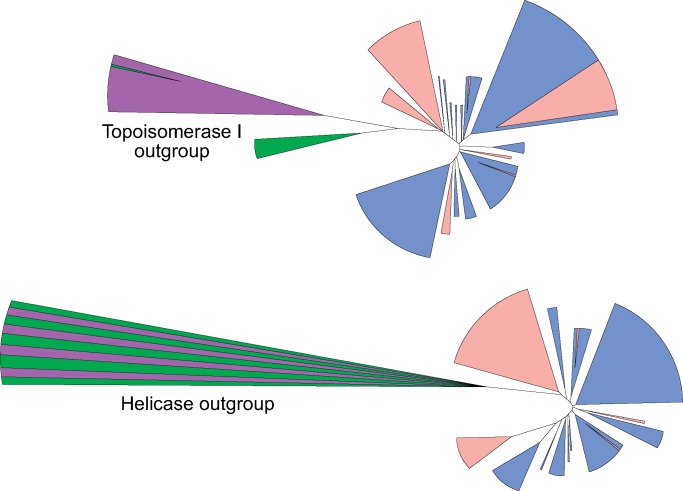
Schematic representation of RG phylogenetic trees rooted using either Topoisomerase I or Helicase sequences. Archaeal outgroup sequences are colored purple, bacterial outgroup sequences in green. Archaeal RG sequences are colored in blue and bacterial RG in red. In both cases, the outgroup is extremely distant from RG and results in different rooting (within the large bacterial clade for Topoisomerase; between a bacterial and archaeal clade for Helicase).

The rooting of the RG tree could potentially bring new arguments in favor of specific scenarios for the origin of RG. If RG initially originated in Archaea the tree should be a priori rooted in the archaeal domain, and similarly for bacteria. RG is thought to have arisen from a gene fusion between a helicase and topoisomerase 1A domain, thus we expect that both helicase- and topoisomerase 1A-containing proteins could act as an appropriate outgroup to root the RG tree. We first used topoisomerase sequences with a known phylogenetic relationship (Forterre et al. 2007) to root the tree. Including these sequences with our data set of RG sequences produces a tree with three well separated clades corresponding to RG, bacterial topoisomerases (orthologues of the *Escherichia* omega-protein), and archaeal topoisomerases (also often annotated as DNA topoisomerase III). In this tree, RG turned out to be rooted in one of the bacterial RG clades (fig. 5, supplementary fig. 11, Supplementary Material online) suggesting that RG may have emerged within an ancestral bacterial group. This bacterial group consists of Thermodesulfobacteria, Aquificae, and Thermotoga species; often represented as deeply branching bacteria (Colman et al. 2016). Interestingly, despite the rooting of RG within a bacterial clade, the archaeal phylogeny is still mostly congruent with canonical archaeal trees, with the exception of the Crenarchaeal paralogues which branch separately (removal of these sequences does not change the position of the root, and greatly improves bootstrap support at basal bipartitions; supplementary fig. 12, Supplementary Material online). This result suggests that RG was indeed present very early in the history of archaea, possibly in LACA, but surprisingly also suggests that RG might have been present early in some Bacteria, and transferred to an ancestral archaeal lineage. It is worth noting that bootstrap support for bipartitions adjacent to the topoisomerase root are weak, and the position of the root was lost when only the Crenarchaeal TopR2-like proteins were removed from the analyses (supplementary fig. 13, Supplementary Material online), with the RG root then falling between a bacterial and archaeal clade. An identical interdomain rooting was found when helicase sequences were used as an outgroup, however in this case although the bootstrap support was higher than that of a topoisomerase rooted tree, the outgroup itself showed polyphyletic archaeal and bacterial sequences (fig. 5, supplementary fig. 14, Supplementary Material online). These results potentially indicate that both topoisomerase and helicase sequences are simply too divergent from RG to act as an appropriate outgroup. If either of these rootings were to be confirmed by future analyses, one could imagine evolutionary scenarios to explain them. For instance, RG could have originated in a subgroup of Bacteria (e.g., an ancestor of Thermodesulfobacteria as in supplementary fig. 11, Supplementary Material online) and later transferred to the archaeal lineage before the emergence of LACA (at the very least, before the divergence of the Crenarchaeota and Euryarchaeota), giving a bacterial rooting. Alternatively if RG originated in the archaeal stem lineage (between the LUCA and the LACA), it could have been transferred from this lineage to a subgroup of Bacteria before the LACA, giving a root between bacterial and archaeal clades as in supplementary figure 14 (Supplementary Material online). Notably, both of these scenarios would imply that the LBCA emerged before the LACA, a possibility which, to our knowledge, has not been considered up to now. However, considering the large distance between RG and the outgroup sequences, and the variability of rooting obtained depending upon the RG data set selected, the above scenarios should be interpreted cautiously.

## Discussion

The work presented here represents the largest data set of RG sequences to date. Phylogenetic analyses of this data set strongly suggests the absence of RG in the LUCA since the archaeal and bacterial RG do not form two monophyletic clades (fig. 2). Indeed, all analyzed tree topologies with monophyletic archaeal and bacterial clades were soundly rejected for the RG data set by a suite of tree topology selection tests. Furthermore, the short branch length between any interdomain clades of our phylogenies (fig. 2) indicates a period of divergence inconsistent with the tempo of evolution between LUCA and the common ancestors of Archaea and Bacteria; the branches between the different archaeal and bacterial clades are all very short, suggesting the existence of a single version of RG. In contrast, using the same set of species, we have shown here that not only do Archaea and Bacteria form two monophyletic clades in phylogenies of markers inferred to be present in LUCA such as EF-G, RNA polymerase, and 16S rRNA, but that the branch between these two clades is very long (fig. 3). It could be argued that the shorter interdomain branches of RG relative to other LUCA proteins simply reflects a higher rate of sequence conservation in RG; however, if true, this requirement for sequence conservation must have been transient (crown branch lengths are similar between RG and the LUCA proteins; fig. 3), and only present during the times where other proteins show the greatest divergence. Such a scenario is at odds with all other proteins inferred to be present in the LUCA; using different species, long branches separating Archaea and Bacteria were also systematically observed in the phylogenies of 36 universal proteins (most likely present in the LUCA) (Da Cunha et al. 2017). Our RG results are in contradiction with recent attempts to reconstruct the proteins of the LUCA (Weiss et al. 2016) (supplementary fig. 1 vs. supplementary fig. 3, Supplementary Material online). This could be explained by the difference in the number of sequences used in the two analyses (97 vs. 376) and the fact that Weiss and colleagues do not include the branch length between the Archaea and Bacteria as a criterion to indicate the presence of a protein in the LUCA. This branch was very short in the RG tree of Weiss and colleagues (supplementary fig. 1, Supplementary Material online) and we could not recover the monophyly of Archaea and Bacteria using their data set, suggesting that the monophyly versus paraphyly of Archaea and Bacteria is sensitive to some parameters of tree reconstruction, further suggesting a nondistinct separation of these usually highly divergent domains.

The absence of RG in LUCA, combined with the apparent requirement of RG for growth at high temperature, suggests the existence of a nonhyperthermophilic LUCA. Although RG knockout strains do appear viable at high temperatures in the laboratory (at least in some species), such strains invariably suffer from temperature-sensitive growth defects (Atomi et al. 2004; Lipscomb et al. 2017; Zhang et al. 2018). Thus the possibility exists that hyperthermophily evolved in the absence of RG, with the subsequent emergence of RG conferring a huge selective advantage such that it rapidly became fixed in all hyperthermophilic lineages. Additionally, we cannot exclude the possibility that another protein served a similar function in the LUCA, conferring a hyperthermophilic growth condition; however, there is no evidence for the existence of such a protein in modern organisms. Thus, this hypothetical protein would have to have been lost in an intermediate mesophilic state of both of the post-LUCA lineages (leading to the LBCA and LACA), or lost in one lineage and replaced with the emergent RG in another. Such a scenario seems unlikely, especially considering the consistency of our RG results with those observed through independent methods. For example, work on ancestral protein and rRNA reconstructions (Galtier et al. 1999; Boussau et al. 2008; Groussin and Gouy 2011) suggest that the LUCA was either a mesophile or a moderate thermophile. Additionally, thermoadaptations observed in membrane lipids (Langworthy and Pond 1986; Wiegel and Michael 2014) and modifications of tRNA (Edmonds et al. 1991; Lorenz et al. 2017) are nonhomologous between bacteria and archaea, suggesting hyperthermophily evolved independently in each lineage rather than being a shared trait from the LUCA. A nonhyperthermophilic LUCA is also in agreement with the idea that LUCA was an organism simpler than modern ones, with smaller ribosomes (Fox 2010) and possibly an RNA genome (Poole and Logan 2005). Indeed, the origin of most DNA replication proteins cannot be traced back to LUCA (Forterre 2002b, 2013), and it seems that RG is not an exception. The transition from a LUCA with an RNA genome to archaea and bacteria with DNA genomes could also explain why the tempo of evolution drastically slowed between LUCA and the two prokaryotic ancestors, considering that DNA can be replicated and repaired much more faithfully than RNA (Forterre 2006). With respect to our RG phylogenies, and RG evolution in general, the short branch lengths between bacterial and archaeal clades would place the emergence of RG in the age of DNA cells, that is, more recently than the time of a rapidly evolving RNA-based LUCA (and post-LUCA lineage). This, perhaps, would seem logical considering the strict DNA substrate-dependence of RG, and RG conferring adaptation to hyperthermophilic growth temperatures—a state likely incompatible with RNA genomes (Ginoza and Zimm 1961). Finally, our work highlights the fact that a widespread distribution across bacterial and archaeal taxa is not sufficient evidence for inferring the presence of a protein in the LUCA. Rather, a clear, well-separated monophyly of Archaea and Bacteria, and deep congruence with canonical phylogenetic relationships should be demonstrated (e.g., those exemplified by RNA polymerase, EF-G, 16S rRNA etc.).

## Materials and Methods

### Generation of RG Data set

The 19 RG sequences available in the SwissProt database (The UniProt Consortium 2017) were downloaded (July 2018) and aligned using MSAProbs v0.9.7 (Liu and Schmidt 2014). The alignment was used to build an HMM representative of confirmed RG proteins, using HMMER v3.1b2 (Eddy 2011) which was subsequently used as a query for an HMM search against the nonredundant protein database (downloaded 17 July 2018).

The presence of helicase-like and topoisomerase-like sequences in our RG HMM (RG is a fusion between a SF2-like helicase domain and a Topoisomerase 1A domain; Confalonieri et al. 1993) resulted in the overwhelming presence of helicase- and topoisomerase-domain containing proteins in our search results, only a subset of which are RG sequences, thus hits were limited by a strict *E*-value cutoff of 10^−100^, and then aligned to identify hits which encode both a helicase and topoisomerase domain in a single amino acid sequence (as per all RG sequences; Confalonieri et al. 1993). Alignments were viewed in Geneious 11.0.4 (https://www.geneious.com; last accessed September 10, 2019). A data set of 371 putative RG sequences was recovered. A second search iteration (using all 371 sequences in generation of the query HMM) did not reveal any new RG sequences, and recovered the entire RG data set.

### Split RG Sequences

Known split RG sequences, for example, those of *M. kandleri* (Kozyavkin et al. 1994) and *Nanoarchaeum equitans* (Capp et al. 2010) had to be added to the data set manually as concatenations. To confirm the nature of split RG sequences, we used the entire output of the HMMer search to generate a simple phylogeny—alignment with ClustalW (Larkin et al. 2007) and tree construction with Fasttree v2.1.9 (Price et al. 2010), both performed on Galaxy@Pasteur (Afgan et al. 2018)—to separate RG-encoding sequences from those of topoisomerase and helicase sequences (supplementary fig. 15, Supplementary Material online). Sequences present in this RG clade, but excluded by our alignment-based hit-refining step were extracted from the tree, and themselves aligned with the 19 Swissprot RG sequences. These sequences indeed included the split RG sequences of the Nanoarchaeota and *Methanopyrus* species as well as truncated sequences (e.g., helicase-domain fragments of *T. maritima* RG used in structural analyses—3OIY, 3P4Y, 3P4X), partial RG sequences recovered from metagenomic studies (e.g., KJR71718 from *Vulcanisaeta sp.* AZ3 and PSO07942 from Candidatus *Marsarchaeota* G2), and potential pseudogenization and/or sequencing errors (e.g., WP_082398367 and WP_082398368 from *Aeropyrum camini* are encoded by two adjacent ORFs overlapping by 4 bp which are out of frame by a single base). Potential new split RG sequences were also recovered in this analysis through visualization of alignments in Geneious 11.0.4 (supplementary fig. 16, Supplementary Material online).

### RG Sequence and Species Analyses

Sequence logos were generated using WebLogo 3.6.0 (Crooks et al. 2004), and structural conservation mapping carried out with ConSurf 2016 (Ashkenazy et al. 2016).

Growth temperatures of RG-encoding organisms were obtained from BacDive (Söhngen et al. 2016) when possible, otherwise original research papers were sourced.

### Phylogenetic Tree Construction

Complete sequences corresponding to these RG hits were downloaded, and aligned with MSAProbs or MAFFT v7.419 (Katoh and Standley 2013). Phylogenetically informative regions were selected using BMGE v1.12 (Criscuolo and Gribaldo 2010) with less strict trimming performed in parallel using Noisy (Dress et al. 2008) to test for artificial shortening of branch lengths (Tan et al. 2015) (supplementary fig. 4 vs. supplementary fig. 17, Supplementary Material online). Substitution models were selected with ModelFinder (Kalyaanamoorthy et al. 2017) and phylogenetic trees were generated using IQ-TREE v1.6.6 (Nguyen et al. 2015). Branch support analysis was performed using ultrafast bootstrap approximation (1,000 replicates) and/or Booster v0.1.2 (100 bootstrap replicates) (Stamatakis 2014). Tests of tree topology were performed in IQ-TREE with 10,000 resamplings using the RELL method. Phylogenies of the complete RG data set were also constructed with RAxML v8.2.11 (Stamatakis 2014) and MrBayes v3.2.6 (Ronquist et al. 2012) in order to confirm the paraphyletic nature of the tree resolved with IQ-TREE (supplementary fig. 18, Supplementary Material online). Trees were visualized with iTol 4.2.3 (Letunic and Bork 2007).

Where phylogenies have concentrated on specific groups and/or particular groups of sequences have been removed from phylogenies, the reduced data sets were realigned, gaps removed, substitution model selected, and trees regenerated. For helicase outgroup generation, sequences were selected from the results of a BLAST search where a selection of RG sequences were used as a query, and results limited to helicase proteins.

Sequence data sets, alignments and tree files are available on Dryad Digital Repository.

## Acknowledgment

This work was funded by the European Research Council under the European Union's Seventh Framework Program (FP/2007-2013)/Project EVOMOBIL - ERC Grant Agreement no. 340440.

## Supplementary Material

Supplementary data are available at *Molecular Biology and Evolution* online.

## Supplementary Material

msz180_Supplementary_Data
