# Supplementary material for: The Evolution of Reverse Gyrase Suggests a Nonhyperthermophilic Last Universal Common Ancestor
Source: Mol Biol Evol. 2019 Sep 3;36(12):2737–47. doi: 10.1093/molbev/msz180 (PMC6878951; doi:10.1093/molbev/msz180)
Supplement: msz180_Supplementary_Data [file msz180_supplementary_data.pdf]

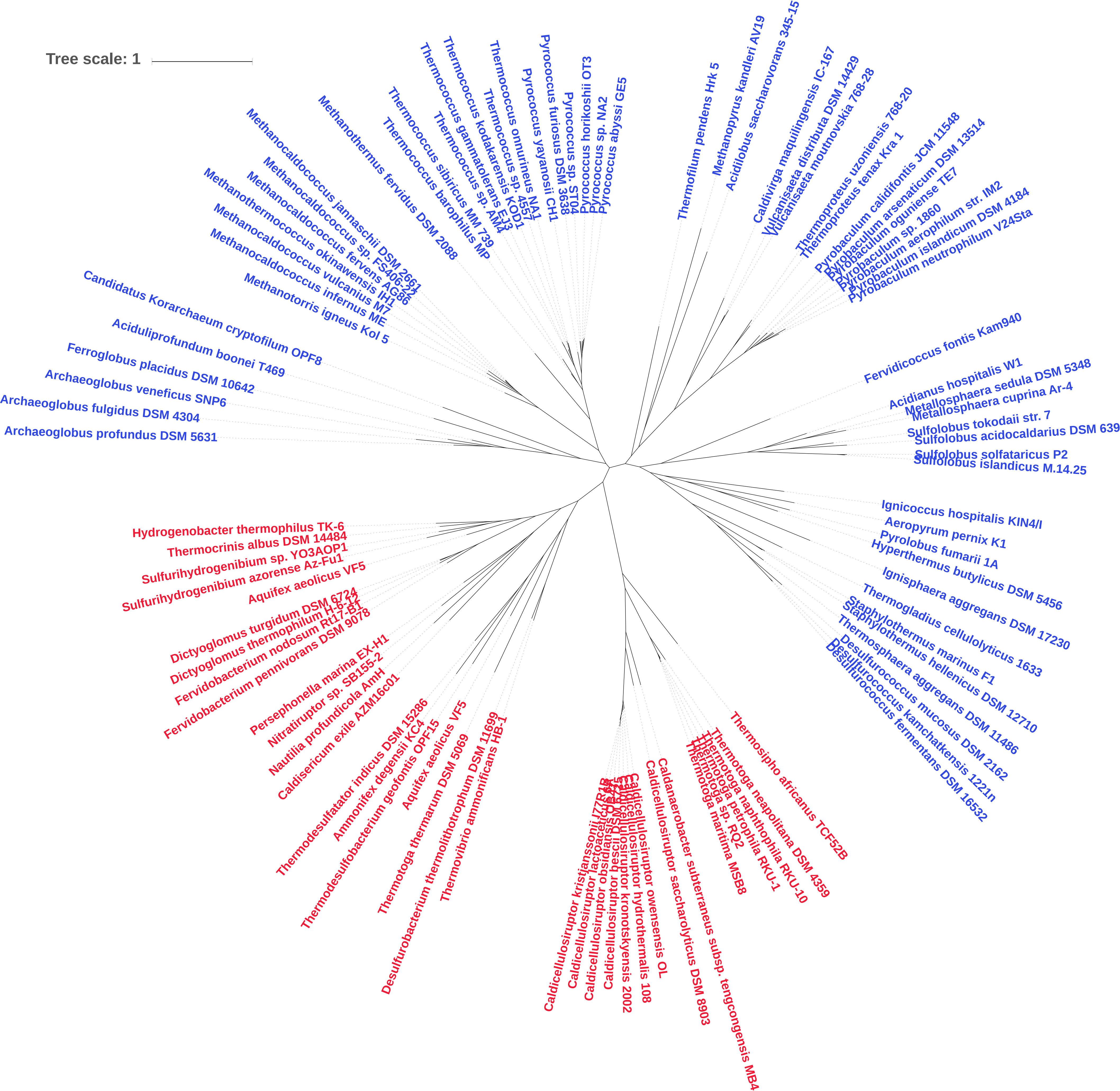

Tree scale: 1
Methanocaldococcus jannaschii DSM 2661
Methanocaldococcus sp. FS406-22
Methanocaldococcus fervens AG86
Methanothermococcus okinawensis IH1
Methanocaldococcus vulcanius M7
Methanocaldococcus infernus ME
Methanotorris igneus Kol 5
Methanothermus fervidus DSM 2088
Thermococcus barophilus MP
Thermococcus sibiricus MM 739
Thermococcus sp. AM4
Thermococcus gammatolerans EJ3
Thermococcus kodakarensis KOD1
Thermococcus sp. 4557
Thermococcus onnurineus NA1
Pyrococcus yayanosii CH1
Pyrococcus furiosus DSM 3638
Pyrococcus sp. ST04
Pyrococcus horikoshii OT3
Pyrococcus sp. NA2
Pyrococcus abyssi GE5
Thermofilum pendens Hrk 5
Methanopyrus kandleri AV19
Acidilobus saccharovorans 345-15
Caldivirga maquilingensis IC-167
Vulcanisaeta distributa DSM 14429
Vulcanisaeta moutnovskia 768-28
Thermoproteus uzoniensis 768-20
Thermoproteus tenax Kra 1
Pyrobaculum calidifontis JCM 11548
Pyrobaculum arsenaticum DSM 13514
Pyrobaculum oguniense TE7
Pyrobaculum sp. 1860
Pyrobaculum aerophilum str. IM2
Pyrobaculum islandicum DSM 4184
Pyrobaculum neutrophilum V24Sta
Fervidicoccus fontis Kam940
Acidianus hospitalis W1
Metallosphaera sedula DSM 5348
Metallosphaera cuprina Ar-4
Sulfolobus tokodaii str. 7
Sulfolobus acidocaldarius DSM 639
Sulfolobus solfataricus P2
Sulfolobus islandicus M.14.25
Ignicoccus hospitalis KIN4/I
Aeropyrum pernix K1
Pyrolobus fumarii 1A
Hyperthermus butylicus DSM 5456
Ignisphaera aggregans DSM 17230
Thermogladius cellulolyticus 1633
Staphylothermus marinus F1
Staphylothermus hellenicus DSM 12710
Thermosphaera aggregans DSM 11486
Desulfurococcus mucosus DSM 2162
Desulfurococcus kamchatkensis 1221n
Desulfurococcus fermentans DSM 16532
Thermosipho africanus TCF52B
Thermotoga neapolitana DSM 4359
Thermotoga naphthophila RKU-10
Thermotoga petrophila RKU-1
Thermotoga sp. RQ2
Thermotoga maritima MSB8
Caldanaerobacter subterraneus subsp. tengcongensis MB4
Caldicellulosiruptor saccharolyticus DSM 8903
Caldicellulosiruptor owensensis OL
Caldicellulosiruptor hydrothermalis 108
Caldicellulosiruptor kronotskyensis 2002
Caldicellulosiruptor bescii DSM
Caldicellulosiruptor obsidiansis
Caldicellulosiruptor lactoaceticus
Caldicellulosiruptor kristjanssonii I77R1B
Thermovibrio ammonificans HB-1
Desulfurobacterium thermolithotrophum DSM 11699
Thermotoga thermarum DSM 5069
Aquifex aeolicus VF5
Thermodesulfobacterium geofontis OPF15
Ammonifex degensii KC4
Thermodesulfatator indicus DSM 15286
Caldisericum exile AZM16c01
Nautilia profundicola AmH
Nitratiruptor sp. SB155-2
Persephonella marina EX-H1
Fervidobacterium pennivorans DSM 9078
Fervidobacterium nodosum Rt17-B1
Dictyoglomus thermophilum H-6-12
Dictyoglomus turgidum DSM 6724
Aquifex aeolicus VF5
Sulfurihydrogenibium azorense Az-Fu1
Sulfurihydrogenibium sp. YO3AOP1
Thermocrinis albus DSM 14484
Hydrogenobacter thermophilus TK-6
Archaeoglobus profundus DSM 5631
Archaeoglobus fulgidus DSM 4304
Archaeoglobus veneficus SNP6
Ferroglobus placidus DSM 10642
Aciduliprofundum boonei T469
Candidatus Korarchaeum cryptofilum OPF8

Supplementary Figure 1. Reproduction of RG phylogeny constructed by Weiss et al. 2016 (1)

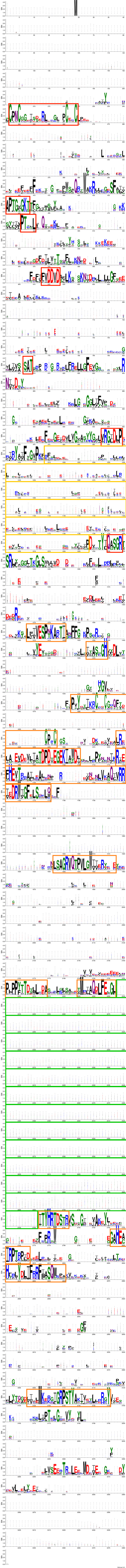

Supplementary Figure 2. Sequence logo representing the alignment of all 376 RG sequences. Regions of interest are highlighted, with conserved helicase motifs (2) in red: position 242-264 Zn finger motif; 441-448 motif I (Walker A); 486-490 motif Ia; 613-618 motif II (Walker B); 807-809 motif III; 993-999 motif V; 1235-1239 motif VI; conserved topoisomerase motifs (3) in orange: position 1408-1420 motif I; 1468-1474 motif 2; 1583-1657 Zn finger motif; 1678-1705 motif 3; 1718-1775 motif 4; 1937-1957 motif 5; 2201-2214 motif 6; 2225-2237 motif 7a; 2732-2742 motif 7b (containing the catalytic tyrosine at position 2734); 2796-2808 motif 8; 2841-2860 motif 9; 3128-3155 motif 10; poorly conserved latch domain indicated in yellow; intein indicated in green.

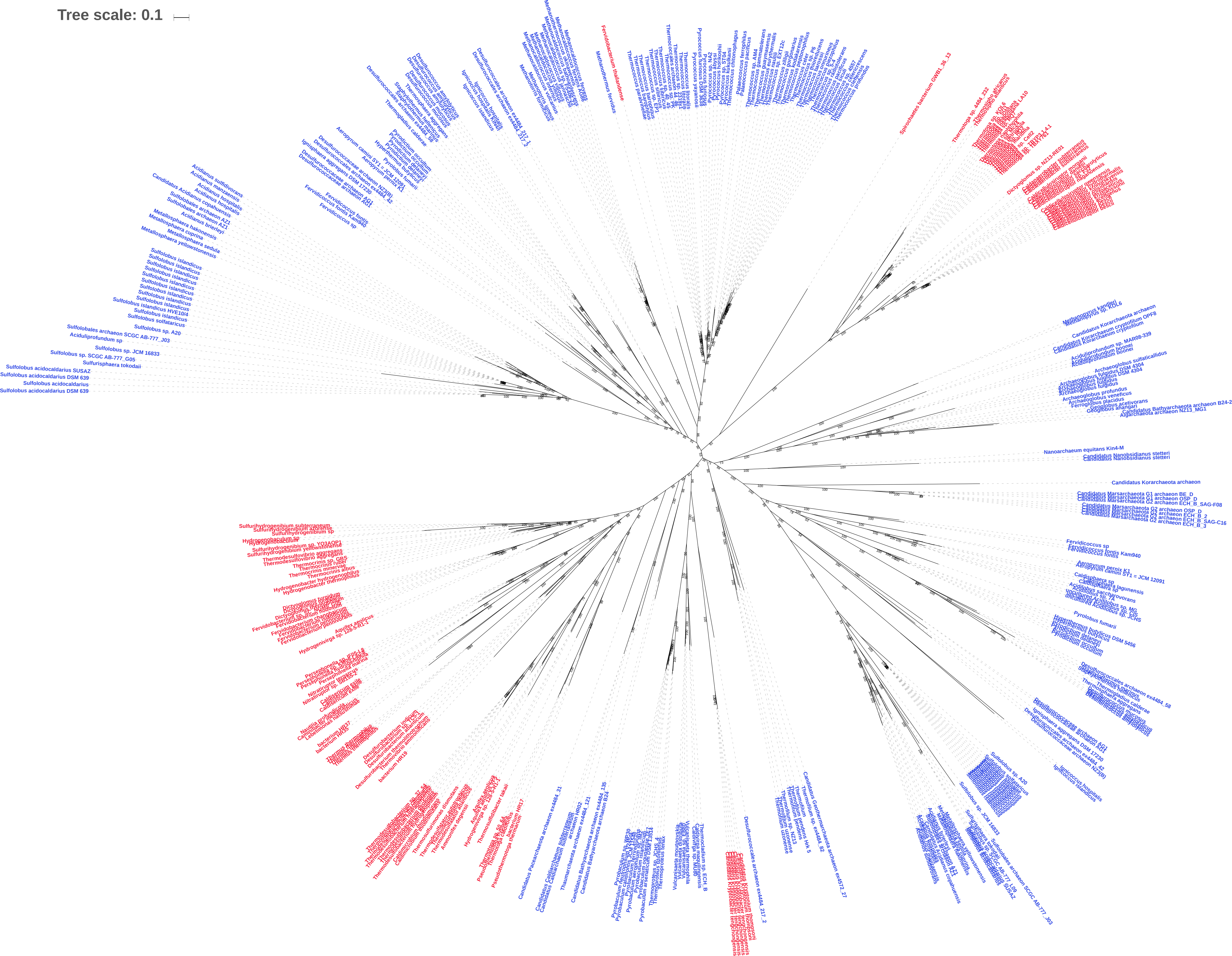
Tree scale: 0.1

Supplementary Figure 3. Phylogenetic reconstruction of entire RG dataset using BMGE for trimming. Branch labels show species source of RG sequence, with Archaea in blue and Bacteria in red. Ultrafast bootstrap values indicated on branches as percentages.

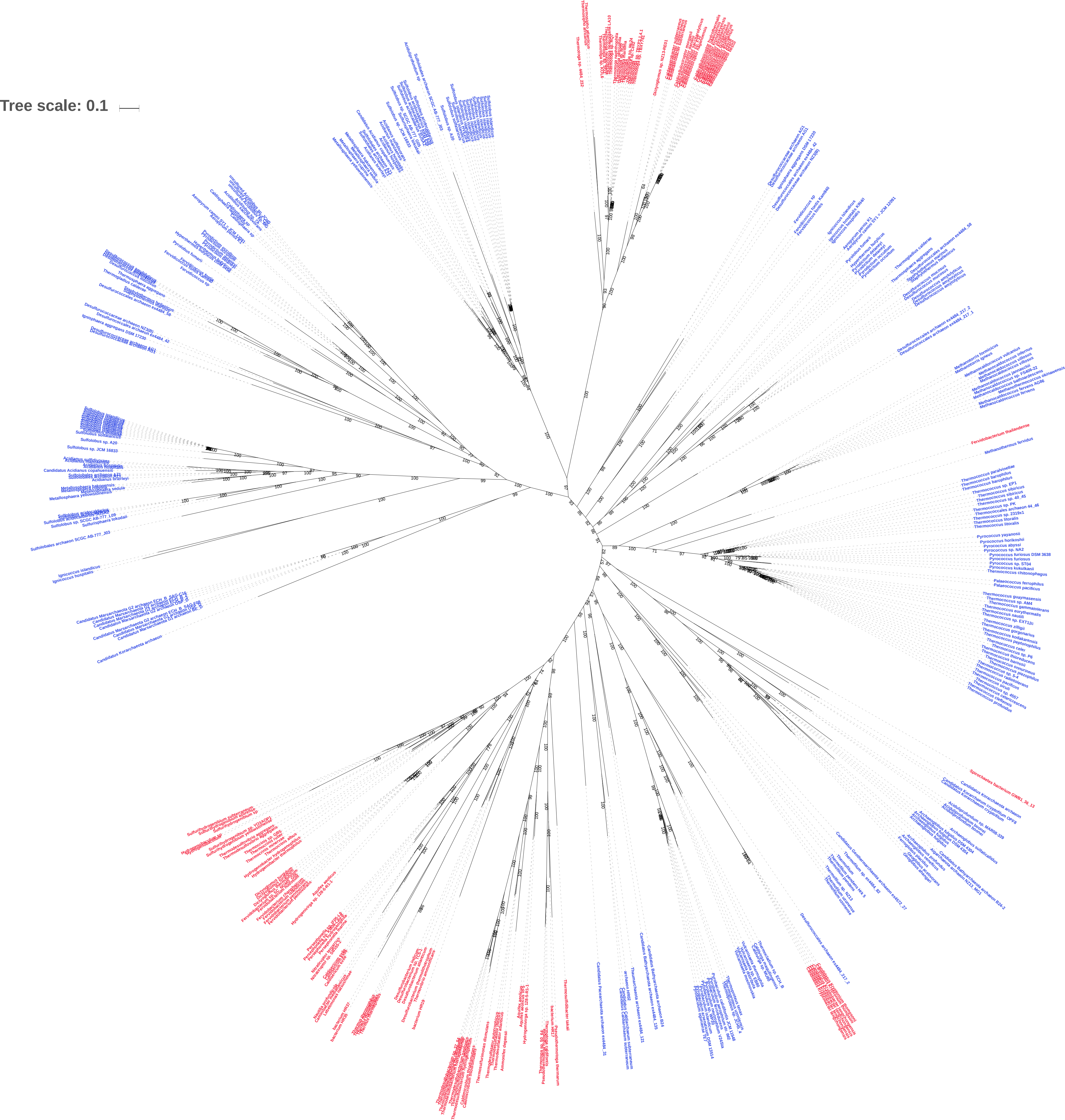
Tree scale: 0.1
Sulfolobales archaeon SCGC AB-777_J03
Ignicoccus islandicus
Ignicoccus hospitalis
Candidatus Korarchaeota archaeon
Sulfolobus sp. A20
Sulfolobus sp. JCM 16833
Candidatus Acidianus copahuensis
Metallosphaera yellowstonensis
Sulfurisphaera tokodaii
Fervidobacterium thailandense
Methanothermus fervidus
Spirochaetes bacterium GWB1_36_13
Desulfurococcales archaeon ex4484_217_2
Desulfurococcales archaeon ex4484_217_1
Thermococcus paralvinellae
Thermococcus barophilus
Thermococcus sibiricus
Thermococcus litoralis
Pyrococcus yayanosii
Pyrococcus horikoshii
Pyrococcus abyssi
Pyrococcus furiosus DSM 3638
Pyrococcus furiosus
Pyrococcus kukulkanii
Thermococcus chitonophagus
Palaeococcus ferrophilus
Palaeococcus pacificus
Thermococcus guaymasensis
Thermococcus gammatolerans
Thermococcus eurythermalis
Thermococcus nautili
Thermococcus zilligii
Thermococcus gorgonarius
Thermococcus kodakarensis
Thermococcus peptonophilus
Thermococcus celer
Thermococcus barossii
Thermococcus onnurineus
Thermococcus piezophilus
Thermococcus profundus
Candidatus Bathyarchaeota archaeon B24
Candidatus Bathyarchaeota archaeon ex4484_135
Thaumarchaeota archaeon ex4484_121
Candidatus Caldiarchaeum subterraneum
Candidatus Pacearchaeota archaeon ex4484_31
Thermosulfidibacter takaii
Pseudothermotoga thermarum
Ammonifex degensii
Thermodesulfovibrio aggregans
Aquifex aeolicus
Hydrogenivirga sp. 128-5-R1-1
Sulfurihydrogenibium subterraneum
Thermotoga sp. 4484_232
Dictyoglomus sp. NZ13-RE01

Supplementary Figure 4. Phylogenetic reconstruction of RG dataset without split-RG sequences from *Methanopyrus* species and Nanoarchaeota. Branch labels show species source of RG sequence, with Archaea in blue and Bacteria in red. Ultrafast bootstrap values indicated on branches as percentages.

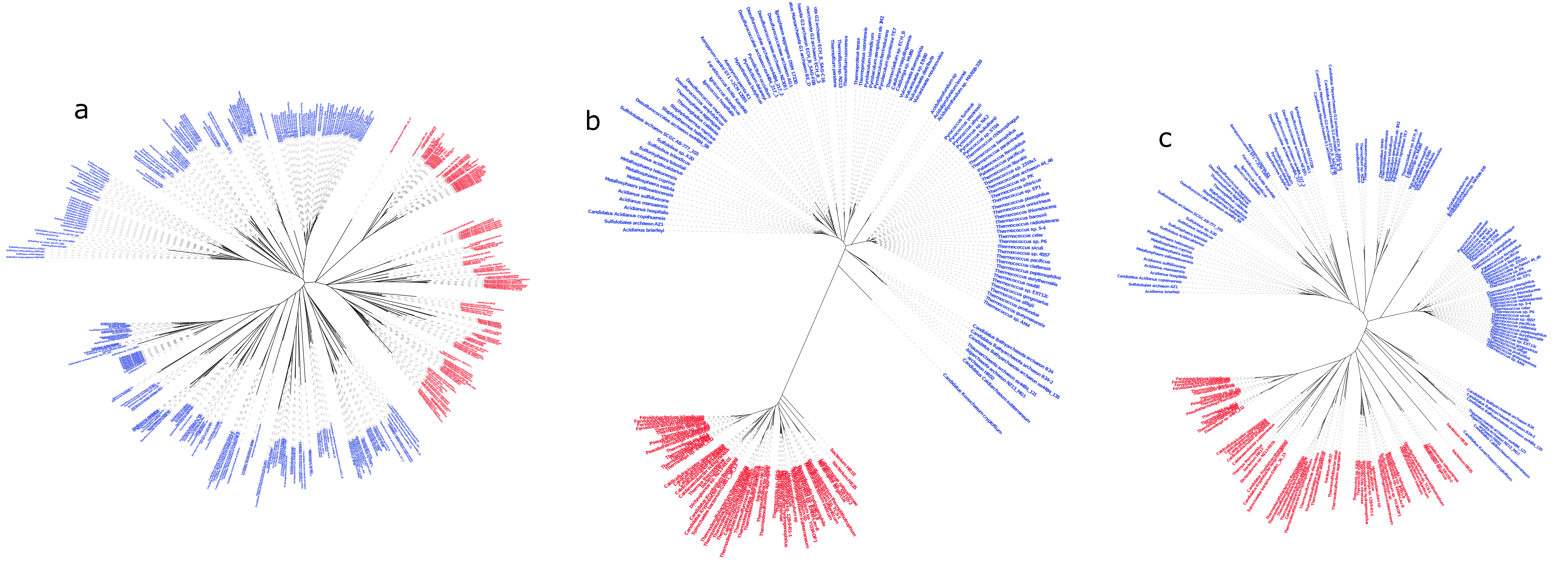

d

	KH	SH	WKH	WSH	ELW	AU
Control tree	1.00	1.00	1.00	1.00	1.00	1.00
Tree a	0.00	0.00	0.00	0.00	9.45e-28	1.24e-41
Tree b	0.00	0.00	0.00	0.00	0.00	1.07e-58
Tree c	0.00	0.00	0.00	0.00	0.00	2.17e-58

Supplementary Figure 5. Experimental tree topologies used for tests of tree selection. a) complete RG phylogeny with 3 bacterial branches moved to produce monophyletic bacterial and archaeal groups; b) tree generated from rpoB sequences from species with identical names to those found in RG tree; c) same tree as in b with artificially shortened inter-domain branch; d) results of tree selection tests - values indicate p-value for exclusion and are colored in green for tree topologies not excluded by the tests, red for tree topologies signficantly excluded. Control tree used in tests is that in Supplementary Figure 3. KH is one-sided Kishino-Hasegawa test; SH is Shimodaira-Hasegawa test; WKH is weighted KH test; WSH is weighted SH test; ELW is Expected Likelihood Weight; AU is approximately unbiased test.

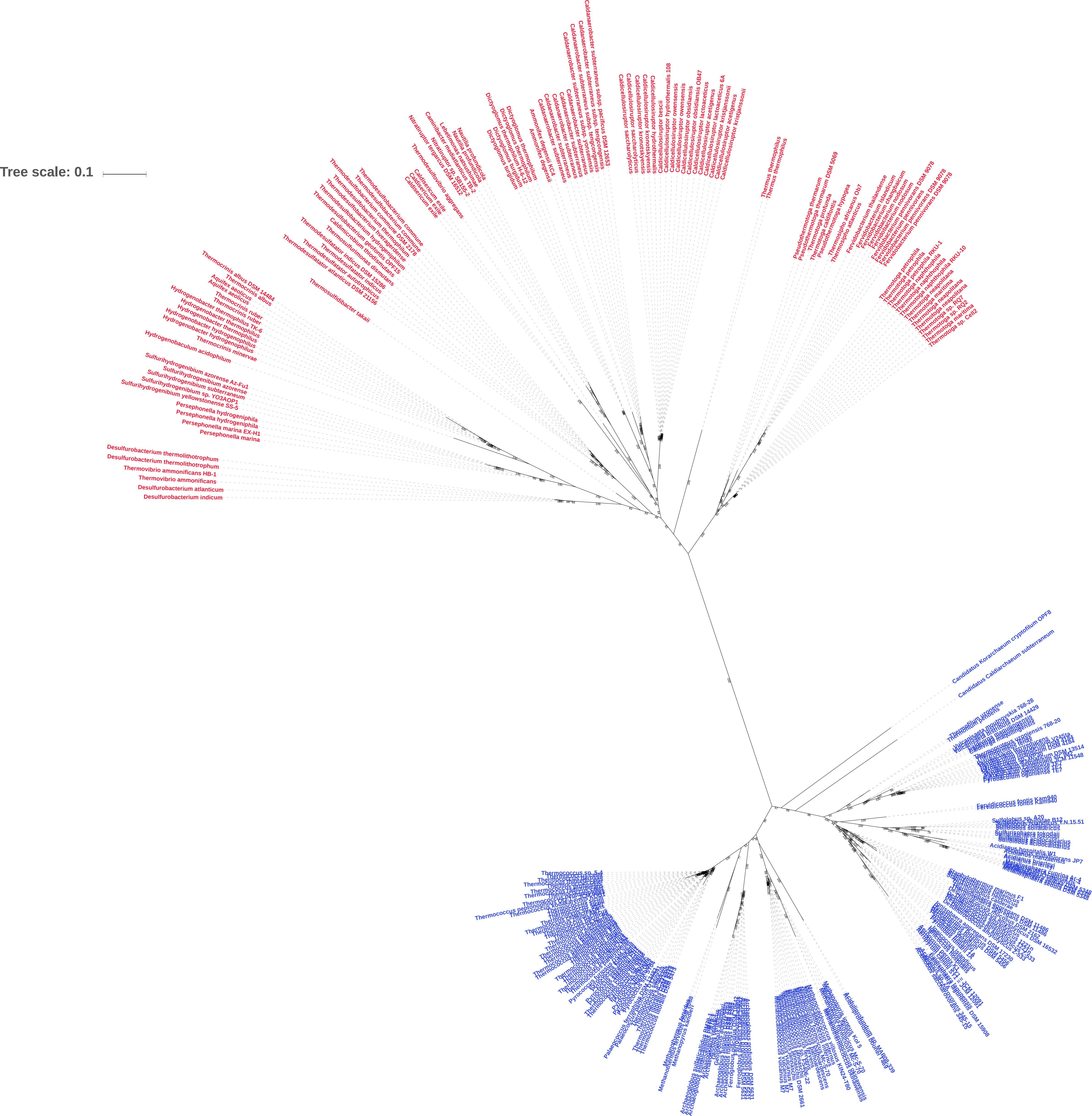
Tree scale: 0.1
Desulfurobacterium thermolithotrophum
Desulfurobacterium thermolithotrophum
Thermovibrio ammonificans HB-1
Thermovibrio ammonificans
Desulfurobacterium atlanticum
Desulfurobacterium indicum
Persephonella marina
Persephonella marina EX-H1
Persephonella hydrogeniphila
Persephonella hydrogeniphila
Sulfurihydrogenibium yellowstonense SS-5
Sulfurihydrogenibium sp. YO3AOP1
Sulfurihydrogenibium subterraneum
Sulfurihydrogenibium azorense
Sulfurihydrogenibium azorense Az-Fu1
Hydrogenobaculum acidophilum
Thermocrinis minervae
Hydrogenobacter hydrogenophilus
Hydrogenobacter hydrogenophilus
Hydrogenobacter thermophilus
Hydrogenobacter thermophilus TK-6
Thermocrinis ruber
Thermocrinis ruber
Aquifex aeolicus
Aquifex aeolicus
Thermocrinis albus
Thermocrinis albus DSM 14484
Thermosulfidibacter takaii
Thermodesulfatator atlanticus DSM 21156
Thermodesulfatator autotrophicus
Thermodesulfatator indicus
Thermodesulfatator indicus DSM 15286
Thermosulfurimonas dismutans
Caldimicrobium thiodismutans
Thermodesulfobacterium geofontis OPF15
Thermodesulfobacterium hydrogeniphilum
Thermodesulfobacterium hveragerdense
Thermodesulfobacterium thermophilum
Thermodesulfobacterium commune DSM 2178
Thermodesulfobacterium commune
Thermodesulfobacterium commune
Caldisericum exile
Caldisericum exile
Caldisericum exile
Thermodesulfovibrio aggregans
Nitratiruptor tergarcus DSM 16512
Nitratiruptor sp. SB155-2
Caminibacter mediatlanticus TB-2
Lebetimonas natsushimae
Nautilia profundicola
Dictyoglomus turgidum
Dictyoglomus turgidum
Dictyoglomus thermophilum H-6-12
Dictyoglomus thermophilum
Ammonifex degensii
Ammonifex degensii KC4
Caldanaerobacter subterraneus
Caldanaerobacter subterraneus subsp. yonseiensis
Caldanaerobacter subterraneus subsp. tengcongensis
Caldanaerobacter subterraneus subsp. pacificus DSM 12653
Caldicellulosiruptor saccharolyticus
Caldicellulosiruptor saccharolyticus
Caldicellulosiruptor kronotskyensis
Caldicellulosiruptor kronotskyensis
Caldicellulosiruptor hydrothermalis
Caldicellulosiruptor hydrothermalis 108
Caldicellulosiruptor bescii
Caldicellulosiruptor owensensis
Caldicellulosiruptor owensensis
Caldicellulosiruptor obsidiansis
Caldicellulosiruptor obsidiansis OB47
Caldicellulosiruptor lactoaceticus
Caldicellulosiruptor acetigenus
Caldicellulosiruptor lactoaceticus 6A
Caldicellulosiruptor acetigenus
Caldicellulosiruptor kristjanssonii
Caldicellulosiruptor kristjanssonii
Thermus thermophilus
Thermus thermophilus
Pseudothermotoga thermarum
Pseudothermotoga thermarum DSM 5069
Thermotoga profunda
Thermotoga caldifontis
Pseudothermotoga hypogea
Thermosipho africanus Ob7
Thermosipho atlanticus
Fervidobacterium thailandense
Fervidobacterium islandicum
Fervidobacterium changbaicum
Fervidobacterium nodosum
Fervidobacterium pennivorans
Fervidobacterium pennivorans DSM 9078
Fervidobacterium pennivorans DSM 9078
Thermotoga petrophila
Thermotoga petrophila RKU-1
Thermotoga naphthophila
Thermotoga naphthophila RKU-10
Thermotoga neapolitana
Thermotoga maritima
Thermotoga neapolitana
Thermotoga sp. RQ7
Thermotoga maritima
Thermotoga sp. RQ2
Thermotoga sp. Cell2
Candidatus Korarchaeum cryptofilum OPF8
Candidatus Caldiarchaeum subterraneum
Fervidicoccus fontis Kam940
Fervidicoccus fontis Kam940
Sulfolobus solfataricus
Sulfurisphaera tokodaii
Acidianus hospitalis W1
Acidianus brierleyi
Thermococcus sp. 5-4
Thermococcus peptonophilus
Palaeococcus ferrophilus
Methanopyrus kandleri

Supplementary Figure 6. Phylogenetic reconstruction of 16S rDNA gene from RG-encoding species. Sequences encoded by Archaeal species are indicated in blue, Bacteria in red.

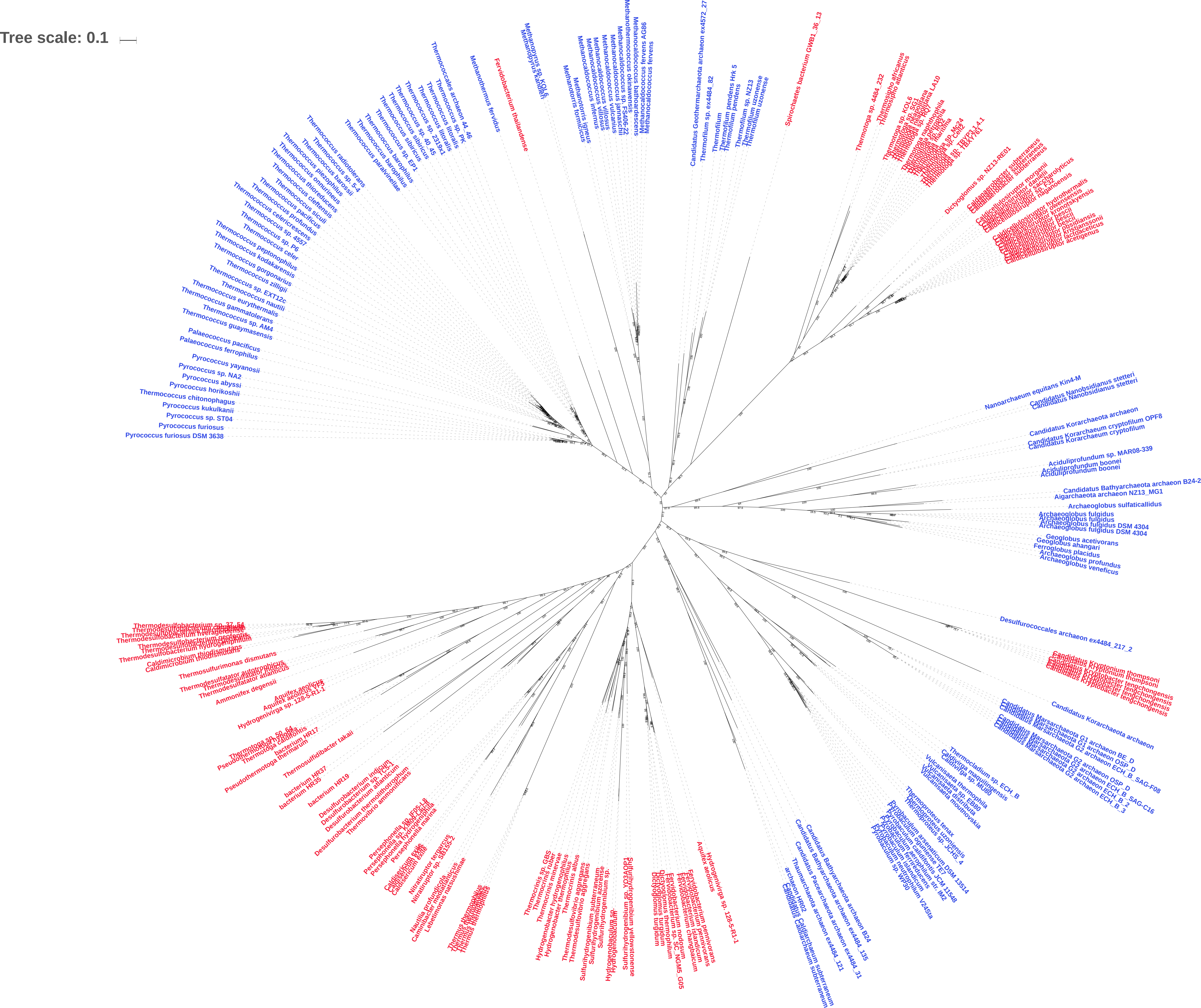

Tree scale: 0.1
Pyrococcus furiosus DSM 3638
Pyrococcus furiosus
Pyrococcus sp. ST04
Pyrococcus kukulkanii
Thermococcus chitonophagus
Pyrococcus horikoshii
Pyrococcus abyssi
Pyrococcus sp. NA2
Pyrococcus yayanosii
Palaeococcus ferrophilus
Palaeococcus pacificus
Thermococcus guaymasensis
Thermococcus sp. AM4
Thermococcus gammatolerans
Thermococcus eurythermalis
Thermococcus nautili
Thermococcus sp. EXT12c
Thermococcus zilligii
Thermococcus gorgonarius
Thermococcus kodakarensis
Thermococcus peptonophilus
Thermococcus sp. P6
Thermococcus celer
Thermococcus sp. 4557
Thermococcus celericrescens
Thermococcus profundus
Thermococcus cleftensis
Thermococcus pacificus
Thermococcus siculi
Thermococcus thioreducens
Thermococcus onnurineus
Thermococcus piezophilus
Thermococcus barossii
Thermococcus sp. 5-4
Thermococcus radiotolerans
Thermococcus paralvinellae
Thermococcus barophilus
Thermococcus sp. EP1
Thermococcus sibiricus
Thermococcus sp. 2319x1
Thermococcus sp. 40_45
Thermococcus litoralis
Thermococcus sp. PK
Thermococcales archaeon 44_46
Methanothermus fervidus
Fervidobacterium thailandense
Methanopyrus sp. KOL6
Methanopyrus kandleri
Methanotorris formicicus
Methanotorris igneus
Methanocaldococcus infernus
Methanocaldococcus villosus
Methanocaldococcus vulcanius
Methanocaldococcus jannaschii
Methanocaldococcus sp. FS406-22
Methanothermococcus okinawensis
Methanocaldococcus bathoardescens
Methanocaldococcus fervens AG86
Methanocaldococcus fervens
Candidatus Geothermarchaeota archaeon ex4572_27
Thermofilum sp. ex4484_82
Thermofilum
Thermofilum pendens Hrk 5
Thermofilum pendens
Thermofilum sp. NZ13
Thermofilum uzonense
Thermofilum uzonense
Spirochaetes bacterium GWB1_36_13
Thermotoga sp. 4484_232
Thermosipho africanus
Thermosipho atlanticus
Dictyoglomus sp. NZ13-RE01
Caldanaerobacter subterraneus
Caldanaerobacter subterraneus
Caldicellulosiruptor morganii
Caldicellulosiruptor danielii
Caldicellulosiruptor saccharolyticus
Caldicellulosiruptor naganoensis
Caldicellulosiruptor hydrothermalis
Caldicellulosiruptor owensensis
Caldicellulosiruptor kronotskyensis
Caldicellulosiruptor bescii
Caldicellulosiruptor obsidiansis
Caldicellulosiruptor kristjanssonii
Caldicellulosiruptor lactoaceticus
Caldicellulosiruptor acetigenus
Nanoarchaeum equitans Kin4-M
Candidatus Nanobsidianus stetteri
Candidatus Nanobsidianus stetteri
Candidatus Korarchaeota archaeon
Candidatus Korarchaeum cryptofilum OPF8
Candidatus Korarchaeum cryptofilum
Aciduliprofundum sp. MAR08-339
Aciduliprofundum boonei
Aciduliprofundum boonei
Candidatus Bathyarchaeota archaeon B24-2
Aigarchaeota archaeon NZ13_MG1
Archaeoglobus sulfaticallidus
Archaeoglobus fulgidus
Archaeoglobus fulgidus
Archaeoglobus fulgidus DSM 4304
Archaeoglobus fulgidus DSM 4304
Geoglobus acetivorans
Geoglobus ahangari
Ferroglobus placidus
Archaeoglobus profundus
Archaeoglobus veneficus
Desulfurococcales archaeon ex4484_217_2
Candidatus Kryptonium thompsoni
Candidatus Kryptonium thompsoni
Candidatus Kryptobacter tengchongensis
Candidatus Kryptobacter tengchongensis
Candidatus Korarchaeota archaeon
Thermocladium sp. ECH_B
Caldivirga maquilingensis
Caldivirga sp. MU80
Vulcanisaeta thermophila
Vulcanisaeta sp. EB80
Vulcanisaeta distributa
Vulcanisaeta moutnovskia
Thermoproteus tenax
Thermoproteus uzoniensis
Thermoproteus sp. CIS_4
Pyrobaculum arsenaticum DSM 13514
Pyrobaculum aerophilum str. IM2
Pyrobaculum calidifontis JCM 11548
Pyrobaculum neutrophilum V24Sta
Pyrobaculum sp. WP30
Candidatus Bathyarchaeota archaeon B24
Candidatus Bathyarchaeota archaeon ex4484_135
Candidatus Pacearchaeota archaeon ex4484_31
Thaumarchaeota archaeon ex4484_121
archaeon HR02
Candidatus Caldiarchaeum subterraneum
Candidatus Caldiarchaeum subterraneum
Hydrogenivirga sp. 128-5-R1-1
Aquifex aeolicus
Fervidobacterium pennivorans
Fervidobacterium islandicum
Fervidobacterium changbaicum
Fervidobacterium nodosum
Fervidobacterium sp. SC_NGM5_G05
Dictyoglomus thermophilum
Dictyoglomus turgidum
Sulfurihydrogenibium yellowstonense
Sulfurihydrogenibium azorense
Sulfurihydrogenibium sp.
Sulfurihydrogenibium subterraneum
Thermodesulfovibrio aggregans
Thermodesulfovibrio aggregans
Thermocrinis albus
Hydrogenobacter thermophilus
Hydrogenobacter hydrogenophilus
Thermocrinis minervae
Thermocrinis ruber
Thermocrinis sp. GBS
Thermus thermophilus
Nautilia profundicola
Caminibacter mediatlanticus
Lebetimonas natsushimae
Nitratiruptor sp. SB155-2
Nitratiruptor tergarcus
Caldisericum exile
Caldisericum exile
Persephonella marina
Persephonella hydrogeniphila
Persephonella sp. KM09-Lau-8
Persephonella sp. IF05-L8
Desulfurobacterium thermolithotrophum
Thermovibrio ammonificans
Desulfurobacterium atlanticum
Desulfurobacterium sp. TC5-1
Desulfurobacterium indicum
bacterium HR19
bacterium HR35
bacterium HR37
Thermosulfidibacter takaii
Pseudothermotoga thermarum
bacterium HR17
Pseudothermotoga hypogea
Thermotoga caldifontis
Thermotoga sp. 50_64
Hydrogenivirga sp. 128-5-R1-1
Aquifex aeolicus VF5
Aquifex aeolicus
Ammonifex degensii
Thermodesulfatator atlanticus
Thermodesulfatator autotrophicus
Thermosulfurimonas dismutans
Caldimicrobium thiodismutans
Caldimicrobium thiodismutans
Thermodesulfobacterium hydrogeniphilum
Thermodesulfobacterium geofontis
Thermodesulfobacterium sp. 37_54

Supplementary Figure 7. Phylogenetic reconstruction of RG dataset with TopR1- and TopR2-like sequences removed. Sequences encoded by Archaeal species are indicated in blue, Bacteria in red. Ultrafast bootstrap values indicated on branches as percentages.

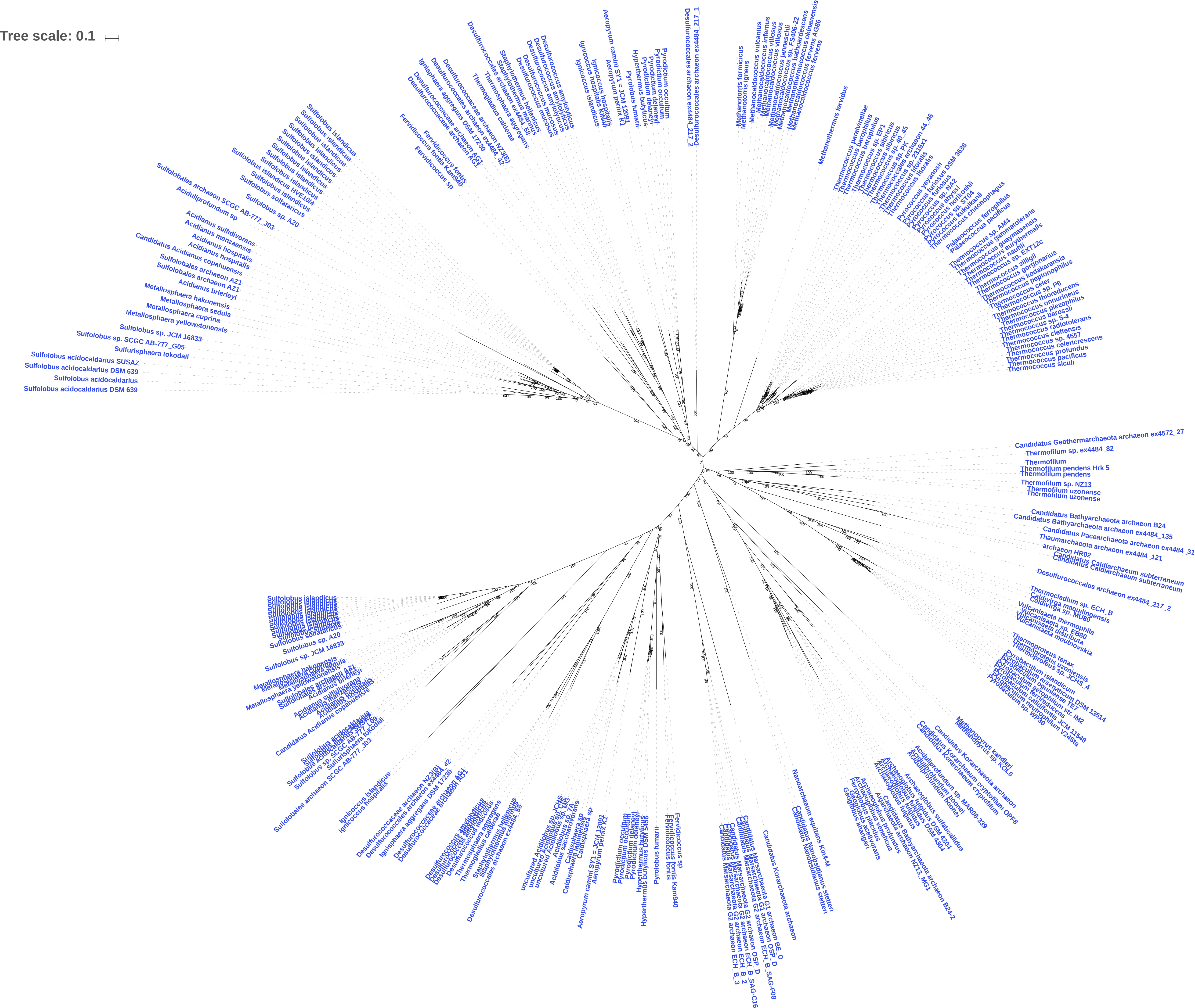

Tree scale: 0.1
Sulfolobus islandicus
Sulfolobus islandicus HVE10/4
Sulfolobus solfataricus
Sulfolobus sp. A20
Sulfolobales archaeon SCGC AB-777_J03
Aciduliprofundum sp
Acidianus sulfidivorans
Acidianus manzaensis
Acidianus hospitalis
Candidatus Acidianus copahuensis
Sulfolobales archaeon AZ1
Acidianus brierleyi
Metallosphaera hakonensis
Metallosphaera sedula
Metallosphaera cuprina
Metallosphaera yellowstonensis
Sulfolobus sp. JCM 16833
Sulfolobus sp. SCGC AB-777_G05
Sulfurisphaera tokodaii
Sulfolobus acidocaldarius SUSAZ
Sulfolobus acidocaldarius DSM 639
Sulfolobus acidocaldarius
Fervidicoccus sp
Fervidicoccus fontis Kam940
Fervidicoccus fontis
Desulfurococcaceae archaeon AG1
Desulfurococcales archaeon AG1
Ignisphaera aggregans DSM 17230
Desulfurococcales archaeon ex4484_42
Desulfurococcaceae archaeon NZ3(B)
Thermogladius calderae
Thermosphaera aggregans
Desulfurococcales archaeon ex4484_58
Staphylothermus hellenicus
Staphylothermus marinus
Desulfurococcus mucosus
Desulfurococcus amylolyticus
Ignicoccus islandicus
Ignicoccus hospitalis
Aeropyrum pernix K1
Aeropyrum camini SY1 = JCM 12091
Pyrolobus fumarii
Hyperthermus butylicus
Pyrodictium delaneyi
Pyrodictium occultum
Desulfurococcales archaeon ex4484_217_1
Desulfurococcales archaeon ex4484_217_2
Methanotorris formicicus
Methanotorris igneus
Methanocaldococcus vulcanius
Methanocaldococcus infernus
Methanocaldococcus villosus
Methanocaldococcus jannaschii
Methanocaldococcus sp. FS406-22
Methanocaldococcus bathoardescens
Methanothermococcus okinawensis
Methanocaldococcus fervens AG86
Methanocaldococcus fervens
Methanothermus fervidus
Thermococcus paralvinellae
Thermococcus barophilus
Thermococcus sp. EP1
Thermococcus sibiricus
Thermococcus sp. 40_45
Thermococcales archaeon 44_46
Thermococcus sp. PK
Thermococcus sp. 2319x1
Thermococcus litoralis
Pyrococcus yayanosii
Pyrococcus furiosus DSM 3638
Pyrococcus furiosus
Pyrococcus sp. NA2
Pyrococcus abyssi
Pyrococcus horikoshii
Pyrococcus sp. ST04
Pyrococcus kukulkanii
Thermococcus chitonophagus
Palaeococcus ferrophilus
Palaeococcus pacificus
Thermococcus sp. AM4
Thermococcus gammatolerans
Thermococcus guaymasensis
Thermococcus eurythermalis
Thermococcus nautili
Thermococcus sp. EXT12c
Thermococcus zilligii
Thermococcus gorgonarius
Thermococcus kodakarensis
Thermococcus peptonophilus
Thermococcus celer
Thermococcus sp. P6
Thermococcus thioreducens
Thermococcus onnurineus
Thermococcus piezophilus
Thermococcus barossii
Thermococcus sp. 5-4
Thermococcus radiotolerans
Thermococcus cleftensis
Thermococcus sp. 4557
Thermococcus celericrescens
Thermococcus profundus
Thermococcus pacificus
Thermococcus siculi
Candidatus Geothermarchaeota archaeon ex4572_27
Thermofilum sp. ex4484_82
Thermofilum
Thermofilum pendens Hrk 5
Thermofilum pendens
Thermofilum sp. NZ13
Thermofilum uzonense
Candidatus Bathyarchaeota archaeon B24
Candidatus Bathyarchaeota archaeon ex4484_135
Candidatus Pacearchaeota archaeon ex4484_31
Thaumarchaeota archaeon ex4484_121
archaeon HR02
Candidatus Caldiarchaeum subterraneum
Desulfurococcales archaeon ex4484_217_2
Thermocladium sp. ECH_B
Caldivirga maquilingensis
Caldivirga sp. MU80
Vulcanisaeta thermophila
Vulcanisaeta sp. EB80
Vulcanisaeta distributa
Vulcanisaeta moutnovskia
Thermoproteus tenax
Thermoproteus uzoniensis
Thermoproteus sp. JCHS_4
Pyrobaculum islandicum DSM 13514
Pyrobaculum arsenaticum
Pyrobaculum aerophilum str. IM2
Pyrobaculum ferrireducens
Pyrobaculum calidifontis JCM 11548
Pyrobaculum neutrophilum V24Sta
Pyrobaculum sp. WP30
Methanopyrus kandleri
Methanopyrus sp. KOL6
Candidatus Korarchaeota archaeon
Candidatus Korarchaeum cryptofilum OPF8
Candidatus Korarchaeum cryptofilum
Aciduliprofundum sp. MAR08-339
Aciduliprofundum boonei
Archaeoglobus sulfaticallidus
Archaeoglobus fulgidus DSM 4304
Candidatus Bathyarchaeota archaeon B24-2
Ferroglobus placidus
Geoglobus acetivorans
Geoglobus ahangari
Nanoarchaeum equitans Kin4-M
Candidatus Nanobsidianus stetteri
Candidatus Korarchaeota archaeon
Candidatus Marsarchaeota G1 archaeon BE_D
Candidatus Marsarchaeota G1 archaeon OSP_D
Candidatus Marsarchaeota G2 archaeon ECH_B_SAG-F08
Candidatus Marsarchaeota G2 archaeon ECH_B_2
Candidatus Marsarchaeota G2 archaeon ECH_B_3
Fervidicoccus fontis Kam940
Pyrolobus fumarii
Hyperthermus butylicus DSM 5456
Aeropyrum camini SY1 = JCM 12091
Aeropyrum pernix K1
Caldisphaera lagunensis
Acidilobus saccharovorans
uncultured Acidilobus sp. JCHS
Desulfurococcales archaeon ex4484_58
Sulfolobales archaeon SCGC AB-777_J03
Ignicoccus islandicus
Ignicoccus hospitalis

Supplementary Figure 8. Phylogenetic reconstruction of complete Archaeal RG dataset. Branch labels show species source of RG sequence. Ultrafast bootstrap values indicated on branches as percentages.

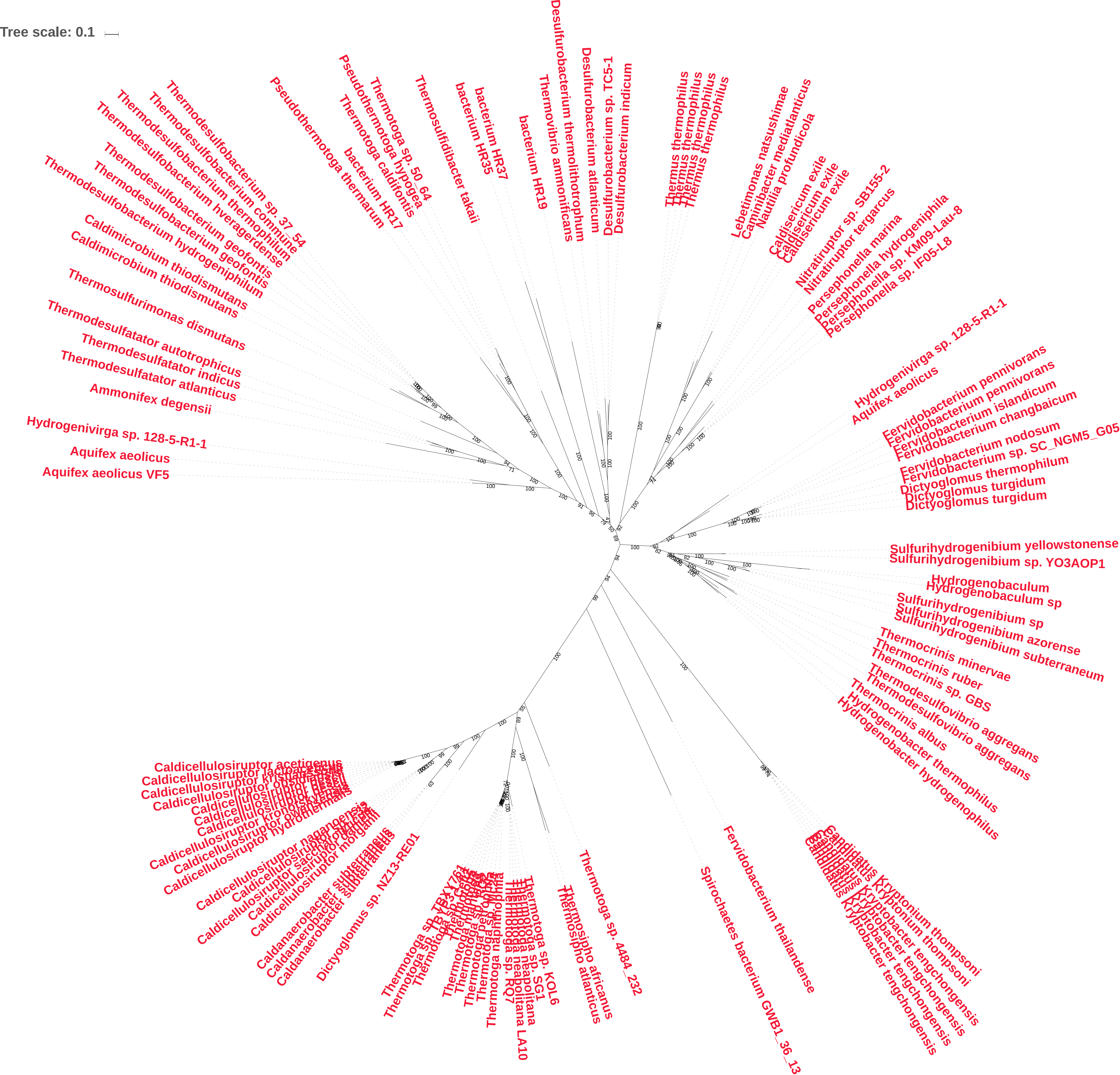

Tree scale: 0.1
Thermodesulfobacterium sp. 37_54
Thermodesulfobacterium commune
Thermodesulfobacterium thermophilum
Thermodesulfobacterium hveragerdense
Thermodesulfobacterium geofontis
Thermodesulfobacterium geofontis
Thermodesulfobacterium hydrogeniphilum
Caldimicrobium thiodismutans
Caldimicrobium thiodismutans
Thermosulfurimonas dismutans
Thermodesulfatator autotrophicus
Thermodesulfatator indicus
Thermodesulfatator atlanticus
Ammonifex degensii
Hydrogenivirga sp. 128-5-R1-1
Aquifex aeolicus
Aquifex aeolicus VF5
Pseudothermotoga thermarum
bacterium HR17
Pseudothermotoga hypogea
Thermotoga caldifontis
Thermotoga sp. 50_64
Thermosulfidibacter takaii
bacterium HR35
bacterium HR37
bacterium HR19
Thermovibrio ammonificans
Desulfurobacterium thermolithotrophum
Desulfurobacterium atlanticum
Desulfurobacterium sp. TC5-1
Desulfurobacterium indicum
Thermus thermophilus
Thermus thermophilus
Thermus thermophilus
Thermus thermophilus
Lebetimonas natsushimae
Caminibacter mediatlanticus
Nautilia profundicola
Caldisericum exile
Caldisericum exile
Caldisericum exile
Nitratiruptor sp. SB155-2
Nitratiruptor tergarcus
Persephonella marina
Persephonella hydrogeniphila
Persephonella sp. KM09-Lau-8
Persephonella sp. IF05-L8
Hydrogenivirga sp. 128-5-R1-1
Aquifex aeolicus
Fervidobacterium pennivorans
Fervidobacterium pennivorans
Fervidobacterium islandicum
Fervidobacterium changbaicum
Fervidobacterium nodosum
Fervidobacterium sp. SC_NGM5_G05
Dictyoglomus thermophilum
Dictyoglomus turgidum
Dictyoglomus turgidum
Sulfurihydrogenibium yellowstonense
Sulfurihydrogenibium sp. YO3AOP1
Hydrogenobaculum
Hydrogenobaculum sp
Sulfurihydrogenibium sp
Sulfurihydrogenibium azorense
Sulfurihydrogenibium subterraneum
Thermocrinis minervae
Thermocrinis ruber
Thermocrinis sp. GBS
Thermodesulfovibrio aggregans
Thermodesulfovibrio aggregans
Thermocrinis albus
Hydrogenobacter thermophilus
Hydrogenobacter hydrogenophilus
Candidatus Kryptonium thompsoni
Candidatus Kryptonium thompsoni
Candidatus Kryptobacter tengchongensis
Fervidobacterium thailandense
Spirochaetes bacterium GWB1_36_13
Thermotoga sp. 4484_232
Thermosipho africanus
Thermosipho atlanticus
Thermotoga sp. KOL6
Thermotoga neapolitana
Thermotoga neapolitana LA10
Thermotoga sp. SG1
Thermotoga sp. RQ7
Thermotoga naphthophila
Dictyoglomus sp. NZ13-RE01
Caldanaerobacter subterraneus
Caldanaerobacter subterraneus
Caldicellulosiruptor acetigenus
Caldicellulosiruptor naganoensis
Caldicellulosiruptor morganii
Caldicellulosiruptor hydrothermalis

Supplementary Figure 9. Phylogenetic reconstruction of complete Bacterial RG dataset. Branch labels show species source of RG sequence. Ultrafast bootstrap values indicated on branches as percentages.

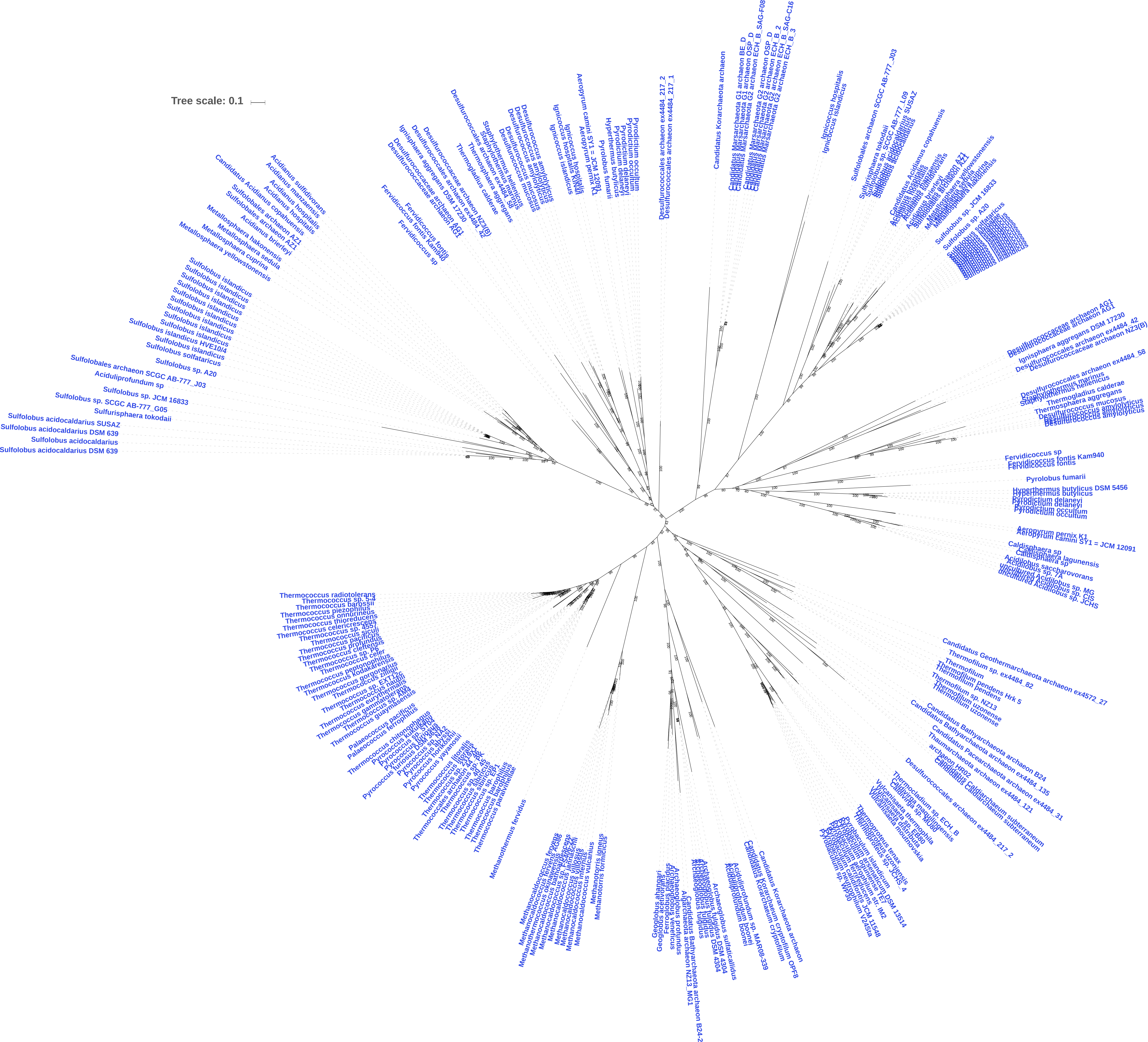
Tree scale: 0.1
Acidianus sulfidivorans
Acidianus manzaensis
Acidianus hospitalis
Candidatus Acidianus copahuensis
Sulfolobales archaeon AZ1
Acidianus brierleyi
Metallosphaera hakonensis
Metallosphaera sedula
Metallosphaera cuprina
Metallosphaera yellowstonensis
Sulfolobus islandicus
Sulfolobus islandicus HVE10/4
Sulfolobus solfataricus
Sulfolobus sp. A20
Sulfolobales archaeon SCGC AB-777_J03
Aciduliprofundum sp
Sulfolobus sp. JCM 16833
Sulfolobus sp. SCGC AB-777_G05
Sulfurisphaera tokodaii
Sulfolobus acidocaldarius SUSAZ
Sulfolobus acidocaldarius DSM 639
Sulfolobus acidocaldarius
Fervidicoccus sp
Fervidicoccus fontis Kam940
Fervidicoccus fontis
Desulfurococcaceae archaeon AG1
Ignisphaera aggregans DSM 17230
Desulfurococcales archaeon ex4484_42
Desulfurococcaceae archaeon NZ3(B)
Desulfurococcales archaeon ex4484_58
Thermogladius calderae
Thermosphaera aggregans
Staphylothermus hellenicus
Staphylothermus marinus
Desulfurococcus mucosus
Desulfurococcus amylolyticus
Ignicoccus islandicus
Ignicoccus hospitalis
Aeropyrum pernix K1
Aeropyrum camini SY1 = JCM 12091
Hyperthermus butylicus
Hyperthermus butylicus DSM 5456
Pyrolobus fumarii
Pyrodictium delaneyi
Pyrodictium occultum
Desulfurococcales archaeon ex4484_217_2
Desulfurococcales archaeon ex4484_217_1
Candidatus Korarchaeota archaeon
Candidatus Marsarchaeota G1 archaeon BE_D
Candidatus Marsarchaeota G1 archaeon OSP_D
Candidatus Marsarchaeota G2 archaeon ECH_B_SAG-F08
Candidatus Marsarchaeota G2 archaeon OSP_D
Candidatus Marsarchaeota G2 archaeon ECH_B_2
Candidatus Marsarchaeota G2 archaeon ECH_B_SAG-C16
Candidatus Marsarchaeota G2 archaeon ECH_B_3
Caldisphaera sp
Caldisphaera lagunensis
Acidilobus saccharovorans
Acidilobus sp. 7A
uncultured Acidilobus sp. MG
uncultured Acidilobus sp. CIS
uncultured Acidilobus sp. JCHS
Candidatus Geothermarchaeota archaeon ex4572_27
Thermofilum sp. ex4484_82
Thermofilum pendens Hrk 5
Thermofilum pendens
Thermofilum sp. NZ13
Thermofilum uzonense
Candidatus Bathyarchaeota archaeon B24
Candidatus Bathyarchaeota archaeon ex4484_135
Candidatus Pacearchaeota archaeon ex4484_31
Thaumarchaeota archaeon ex4484_121
Candidatus Caldiarchaeum subterraneum
Thermocladium sp. ECH_B
Caldivirga maquilingensis
Vulcanisaeta thermophila
Vulcanisaeta souniana
Vulcanisaeta distributa
Vulcanisaeta moutnovskia
Thermoproteus tenax
Thermoproteus uzoniensis
Pyrobaculum islandicum
Pyrobaculum arsenaticum
Pyrobaculum aerophilum str. IM2
Pyrobaculum calidifontis JCM 11548
Pyrobaculum oguniense TE7
Pyrobaculum sp. WP30
Pyrobaculum neutrophilum V24Sta
Candidatus Korarchaeum cryptofilum OPF8
Aciduliprofundum sp. MAR08-339
Aciduliprofundum boonei
Archaeoglobus sulfaticallidus
Archaeoglobus fulgidus DSM 4304
Archaeoglobus fulgidus
Candidatus Bathyarchaeota archaeon B24-2
Aigarchaeota archaeon NZ13_MG1
Archaeoglobus profundus
Archaeoglobus veneficus
Ferroglobus placidus
Geoglobus acetivorans
Geoglobus ahangari
Methanotorris formicicus
Methanotorris igneus
Methanocaldococcus vulcanius
Methanocaldococcus jannaschii
Methanocaldococcus fervens
Methanothermus fervidus
Thermococcus radiotolerans
Thermococcus sp. 5-4
Thermococcus barossii
Thermococcus piezophilus
Thermococcus onnurineus
Thermococcus thioreducens
Thermococcus celericrescens
Thermococcus sp. 4557
Thermococcus siculi
Thermococcus pacificus
Thermococcus profundus
Thermococcus cleftensis
Thermococcus sp. P6
Thermococcus celer
Thermococcus peptonophilus
Thermococcus kodakarensis
Thermococcus gorgonarius
Thermococcus zilligii
Thermococcus sp. EXT12c
Thermococcus nautili
Thermococcus eurythermalis
Thermococcus gammatolerans
Thermococcus sp. AM4
Thermococcus guaymasensis
Palaeococcus pacificus
Palaeococcus ferrophilus
Thermococcus chitonophagus
Pyrococcus kukulkanii
Pyrococcus sp. ST04
Pyrococcus furiosus
Pyrococcus horikoshii
Pyrococcus yayanosii
Thermococcus litoralis
Thermococcus barophilus
Thermococcus paralvinellae

Supplementary Figure 10. Phylogenetic reconstruction of complete Archaeal RG dataset with split-RG sequences removed. Branch labels show species source of RG sequence. Ultrafast bootstrap values indicated on branches as percentages.

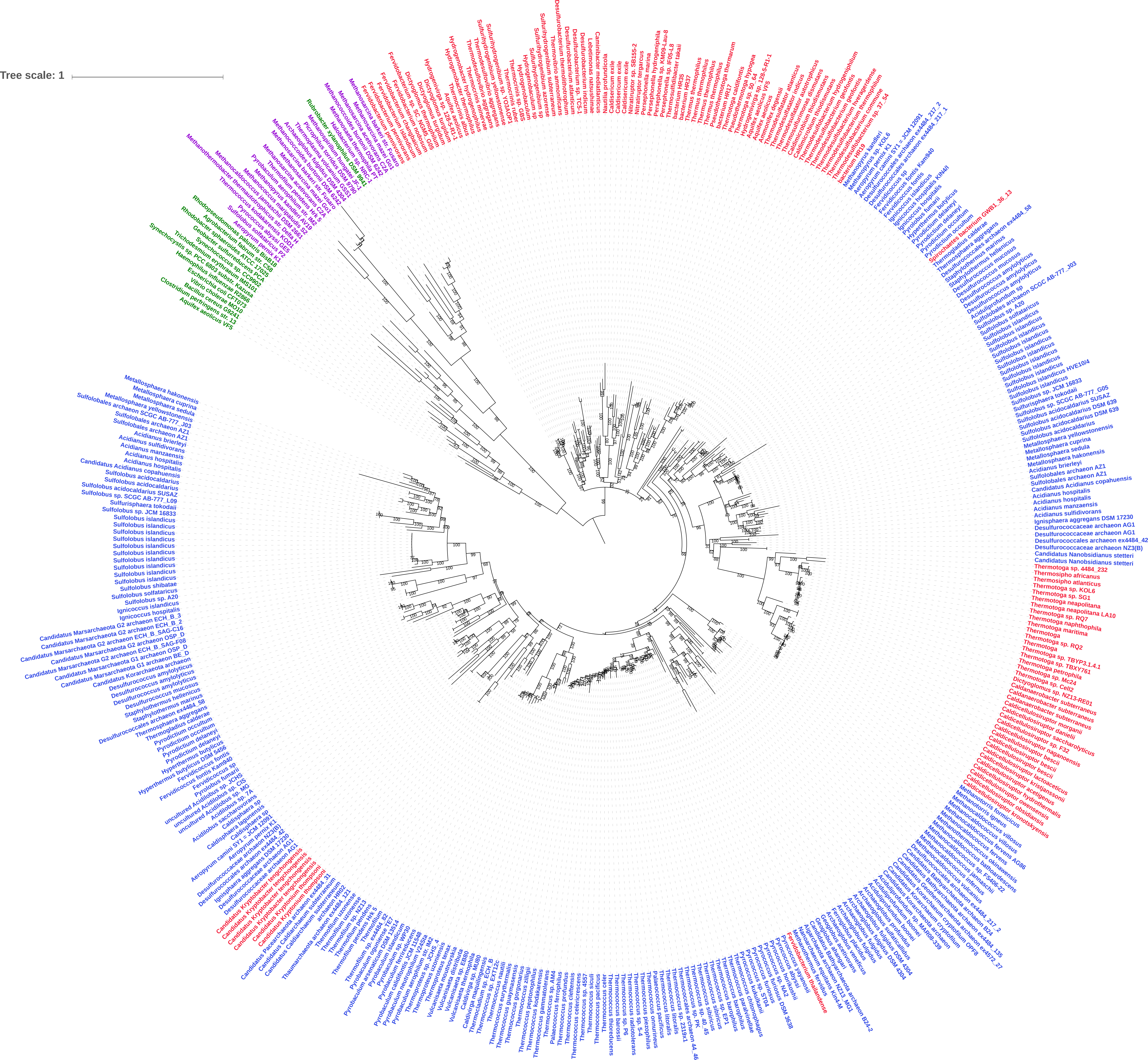
Tree scale: 1

Supplementary Figure 11. Phylogenetic reconstruction of RG dataset. Bacterial and Archaeal topoisomerase sequences included as an outgroup to root the tree. Branch labels show species source of RG sequence, with Archaea in blue and Bacteria in red; or topoisomerase source, with Archaea in purple, Bacteria in green. Ultrafast bootstrap values indicated on branches as percentages.

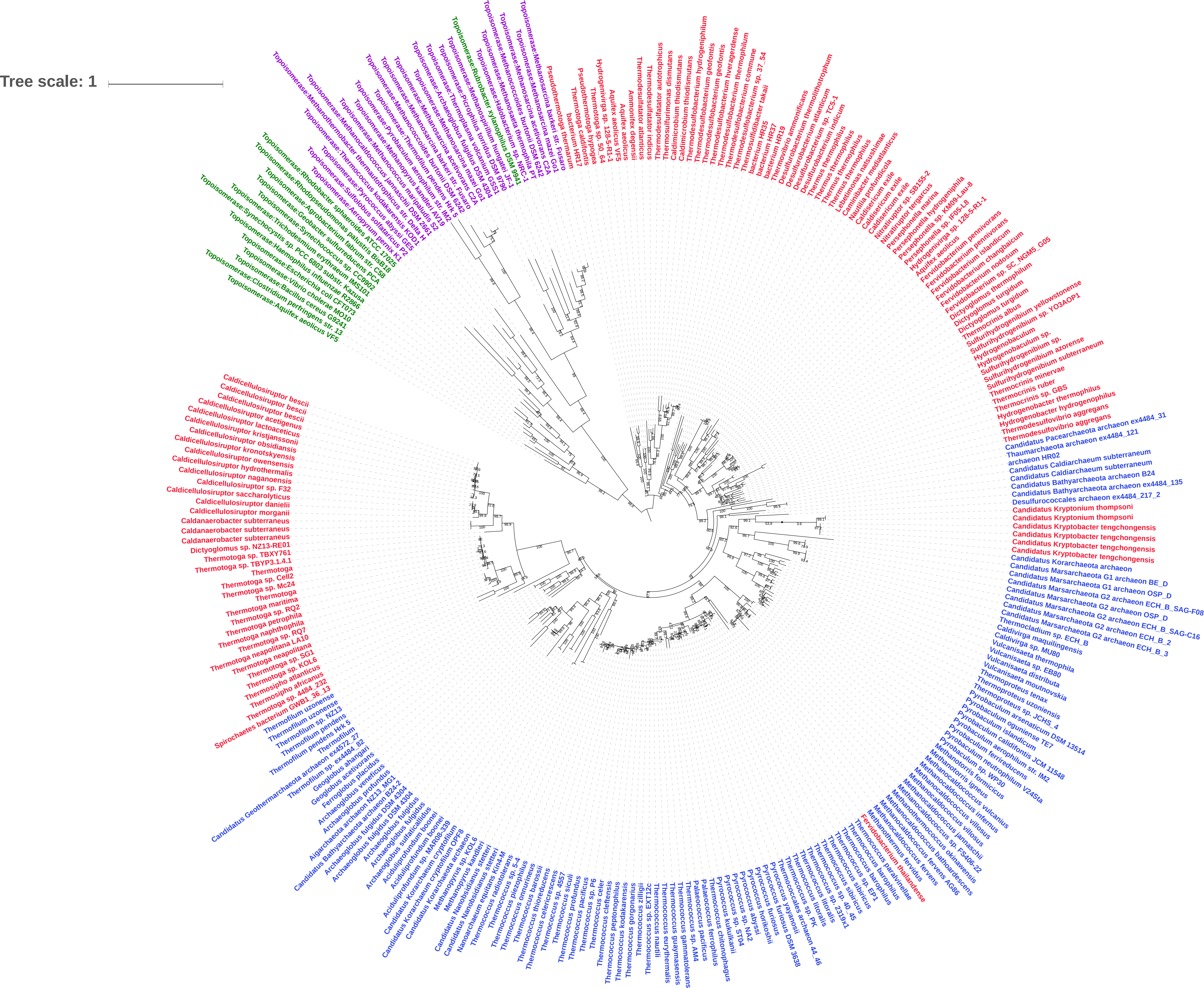
Tree scale: 1
Topoisomerase:Aquifex aeolicus VF5
Topoisomerase:Clostridium perfringens str. 13
Topoisomerase:Bacillus cereus G9241
Topoisomerase:Vibrio cholerae MO10
Topoisomerase:Escherichia coli CFT073
Topoisomerase:Haemophilus influenzae R2866
Topoisomerase:Synechocystis sp. PCC 6803 substr. Kazusa
Topoisomerase:Trichodesmium erythraeum IMS101
Topoisomerase:Synechococcus sp. CC9902
Topoisomerase:Geobacter sulfurreducens PCA
Topoisomerase:Agrobacterium fabrum str. C58
Topoisomerase:Rhodopseudomonas palustris BisB18
Topoisomerase:Rhodobacter sphaeroides ATCC 17025
Topoisomerase:Aeropyrum pernix K1
Topoisomerase:Sulfolobus solfataricus P2
Topoisomerase:Pyrococcus abyssi GE5
Topoisomerase:Thermococcus kodakarensis KOD1
Topoisomerase:Methanothermobacter thermautotrophicus str. Delta H
Topoisomerase:Methanocaldococcus jannaschii DSM 2661
Topoisomerase:Methanococcus maripaludis S2
Topoisomerase:Methanopyrus kandleri AV19
Topoisomerase:Pyrobaculum aerophilum str. IM2
Topoisomerase:Thermofilum pendens Hrk 5
Topoisomerase:Methanococcoides burtonii DSM 6242
Topoisomerase:Methanosarcina barkeri str. Fusaro
Topoisomerase:Methanosarcina acetivorans C2A
Topoisomerase:Methanosarcina mazei Go1
Topoisomerase:Archaeoglobus fulgidus DSM 4304
Topoisomerase:Thermoplasma volcanium GSS1
Topoisomerase:Picrophilus torridus DSM 9790
Topoisomerase:Methanospirillum hungatei JF-1
Topoisomerase:Rubrobacter xylanophilus DSM 9941
Topoisomerase:Halobacterium sp. NRC-1
Topoisomerase:Methanosaeta thermophila PT
Topoisomerase:Methanococcoides burtonii DSM 6242
Topoisomerase:Methanosarcina acetivorans C2A
Topoisomerase:Methanosarcina mazei Go1
Topoisomerase:Methanosarcina barkeri str. Fusaro
Pseudothermotoga thermarum
bacterium HR17
Thermotoga caldifontis
Pseudothermotoga hypogea
Hydrogenivirga sp. 128-5-R1-1
Thermotoga sp. 50_64
Aquifex aeolicus VF5
Aquifex aeolicus
Ammonifex degensii
Thermodesulfatator atlanticus
Thermodesulfatator indicus
Thermodesulfatator autotrophicus
Thermosulfurimonas dismutans
Caldimicrobium thiodismutans
Caldimicrobium thiodismutans
Thermodesulfobacterium hydrogeniphilum
Thermodesulfobacterium geofontis
Thermodesulfobacterium geofontis
Thermodesulfobacterium hveragerdense
Thermodesulfobacterium thermophilum
Thermodesulfobacterium commune
Thermodesulfobacterium sp. 37_54
Thermosulfidibacter takaii
bacterium HR35
bacterium HR37
bacterium HR19
Thermovibrio ammonificans
Desulfurobacterium thermolithotrophum
Desulfurobacterium atlanticum
Desulfurobacterium sp. TC5-1
Desulfurobacterium indicum
Thermus thermophilus
Thermus thermophilus
Thermus thermophilus
Thermus thermophilus
Lebetimonas natsushimae
Caminibacter mediatlanticus
Nautilia profundicola
Caldisericum exile
Caldisericum exile
Nitratiruptor sp. SB155-2
Nitratiruptor tergarcus
Persephonella marina
Persephonella hydrogeniphila
Persephonella sp. KM09-Lau-8
Persephonella sp. IF05-L8
Hydrogenivirga sp. 128-5-R1-1
Aquifex aeolicus
Fervidobacterium pennivorans
Fervidobacterium pennivorans
Fervidobacterium islandicum
Fervidobacterium changbaicum
Fervidobacterium nodosum
Fervidobacterium sp. SC_NGM5_G05
Dictyoglomus thermophilum
Dictyoglomus turgidum
Dictyoglomus turgidum
Thermocrinis albus
Sulfurihydrogenibium yellowstonense
Sulfurihydrogenibium sp. YO3AOP1
Hydrogenobaculum
Hydrogenobaculum sp.
Sulfurihydrogenibium sp.
Sulfurihydrogenibium azorense
Sulfurihydrogenibium subterraneum
Thermocrinis minervae
Thermocrinis ruber
Thermocrinis sp. GBS
Hydrogenobacter thermophilus
Hydrogenobacter hydrogenophilus
Thermodesulfovibrio aggregans
Thermodesulfovibrio aggregans
Candidatus Pacearchaeota archaeon ex4484_31
Thaumarchaeota archaeon ex4484_121
archaeon HR02
Candidatus Caldiarchaeum subterraneum
Candidatus Caldiarchaeum subterraneum
Candidatus Bathyarchaeota archaeon B24
Candidatus Bathyarchaeota archaeon ex4484_135
Desulfurococcales archaeon ex4484_217_2
Candidatus Kryptonium thompsoni
Candidatus Kryptonium thompsoni
Candidatus Kryptobacter tengchongensis
Candidatus Kryptobacter tengchongensis
Candidatus Kryptobacter tengchongensis
Candidatus Kryptobacter tengchongensis
Candidatus Korarchaeota archaeon
Candidatus Marsarchaeota G1 archaeon BE_D
Candidatus Marsarchaeota G1 archaeon OSP_D
Candidatus Marsarchaeota G2 archaeon ECH_B_SAG-F08
Candidatus Marsarchaeota G2 archaeon OSP_D
Candidatus Marsarchaeota G2 archaeon ECH_B_SAG-C16
Candidatus Marsarchaeota G2 archaeon ECH_B_2
Candidatus Marsarchaeota G2 archaeon ECH_B_3
Thermocladium sp. ECH_B
Caldivirga maquilingensis
Caldivirga sp. MU80
Vulcanisaeta thermophila
Vulcanisaeta sp. EB80
Vulcanisaeta distributa
Vulcanisaeta moutnovskia
Thermoproteus tenax
Thermoproteus uzoniensis
Thermoproteus sp. JCHS_4
Pyrobaculum arsenaticum DSM 13514
Pyrobaculum oguniense TE7
Pyrobaculum islandicum
Pyrobaculum calidifontis JCM 11548
Pyrobaculum aerophilum str. IM2
Pyrobaculum ferrireducens
Pyrobaculum neutrophilum V24Sta
Pyrobaculum sp. WP30
Methanotorris formicicus
Methanotorris igneus
Methanocaldococcus vulcanius
Methanocaldococcus infernus
Methanocaldococcus villosus
Methanocaldococcus villosus
Methanocaldococcus jannaschii
Methanocaldococcus sp. FS406-22
Methanothermococcus okinawensis
Methanocaldococcus bathoardescens
Methanocaldococcus fervens AG86
Methanocaldococcus fervens
Methanothermus fervidus
Fervidobacterium thailandense
Thermococcus paralvinellae
Thermococcus barophilus
Thermococcus barophilus
Thermococcus sp. EP1
Thermococcus sibiricus
Thermococcus sibiricus
Thermococcus sp. 40_45
Thermococcus sp. 2319x1
Thermococcus litoralis
Thermococcus litoralis
Thermococcus sp. PK
Thermococcales archaeon 44_46
Pyrococcus yayanosii
Pyrococcus furiosus DSM 3638
Pyrococcus furiosus
Pyrococcus horikoshii
Pyrococcus abyssi
Pyrococcus sp. NA2
Pyrococcus sp. ST04
Pyrococcus kukulkanii
Thermococcus chitonophagus
Thermococcus ferrophilus
Palaeococcus ferrophilus
Palaeococcus pacificus
Thermococcus sp. AM4
Thermococcus gammatolerans
Thermococcus guaymasensis
Thermococcus eurythermalis
Thermococcus nautili
Thermococcus sp. EXT12c
Thermococcus zilligii
Thermococcus gorgonarius
Thermococcus kodakarensis
Thermococcus peptonophilus
Thermococcus cleftensis
Thermococcus celer
Thermococcus sp. P6
Thermococcus pacificus
Thermococcus profundus
Thermococcus siculi
Thermococcus sp. 4557
Thermococcus celericrescens
Thermococcus thioreducens
Thermococcus barossii
Thermococcus onnurineus
Thermococcus piezophilus
Thermococcus sp. 5-4
Thermococcus radiotolerans
Nanoarchaeum equitans Kin4-M
Candidatus Nanobsidianus stetteri
Candidatus Nanobsidianus stetteri
Methanopyrus kandleri
Methanopyrus sp. KOL6
Candidatus Korarchaeota archaeon
Candidatus Korarchaeum cryptofilum OPF8
Candidatus Korarchaeum cryptofilum
Aciduliprofundum sp. MAR08-339
Aciduliprofundum boonei
Aciduliprofundum boonei
Archaeoglobus sulfaticallidus
Archaeoglobus fulgidus
Archaeoglobus fulgidus
Archaeoglobus fulgidus DSM 4304
Archaeoglobus fulgidus DSM 4304
Candidatus Bathyarchaeota archaeon B24-2
Aigarchaeota archaeon NZ13_MG1
Archaeoglobus profundus
Archaeoglobus veneficus
Ferroglobus placidus
Geoglobus acetivorans
Geoglobus ahangari
Thermofilum sp. ex4484_82
Candidatus Geothermarchaeota archaeon ex4572_27
Thermofilum
Thermofilum pendens Hrk 5
Thermofilum pendens
Thermofilum sp. NZ13
Thermofilum uzonense
Thermofilum uzonense
Spirochaetes bacterium GWB1_36_13
Thermotoga sp. 4484_232
Thermosipho africanus
Thermosipho atlanticus
Thermotoga sp. KOL6
Thermotoga sp. SG1
Thermotoga neapolitana
Thermotoga neapolitana LA10
Thermotoga sp. RQ7
Thermotoga naphthophila
Thermotoga petrophila
Thermotoga sp. RQ2
Thermotoga maritima
Thermotoga
Thermotoga sp. Mc24
Thermotoga sp. Cell2
Thermotoga
Thermotoga sp. TBYP3.1.4.1
Thermotoga sp. TBXY761
Dictyoglomus sp. NZ13-RE01
Caldanaerobacter subterraneus
Caldanaerobacter subterraneus
Caldanaerobacter subterraneus
Caldicellulosiruptor morganii
Caldicellulosiruptor danielii
Caldicellulosiruptor saccharolyticus
Caldicellulosiruptor sp. F32
Caldicellulosiruptor naganoensis
Caldicellulosiruptor hydrothermalis
Caldicellulosiruptor owensensis
Caldicellulosiruptor kronotskyensis
Caldicellulosiruptor obsidiansis
Caldicellulosiruptor kristjanssonii
Caldicellulosiruptor lactoaceticus
Caldicellulosiruptor acetigenus
Caldicellulosiruptor bescii
Caldicellulosiruptor bescii
Caldicellulosiruptor bescii

Supplementary Figure 12. Phylogenetic reconstruction of RG dataset with TopR1- and TopR2-like paralogues removed. Bacterial and Archaeal topoisomerase sequences included as an outgroup to root the tree. Branch labels show species source of RG sequence, with Archaea in blue and Bacteria in red; or topoisomerase source, with Archaea in purple, Bacteria in green. Ultrafast bootstrap values indicated on branches as percentages.

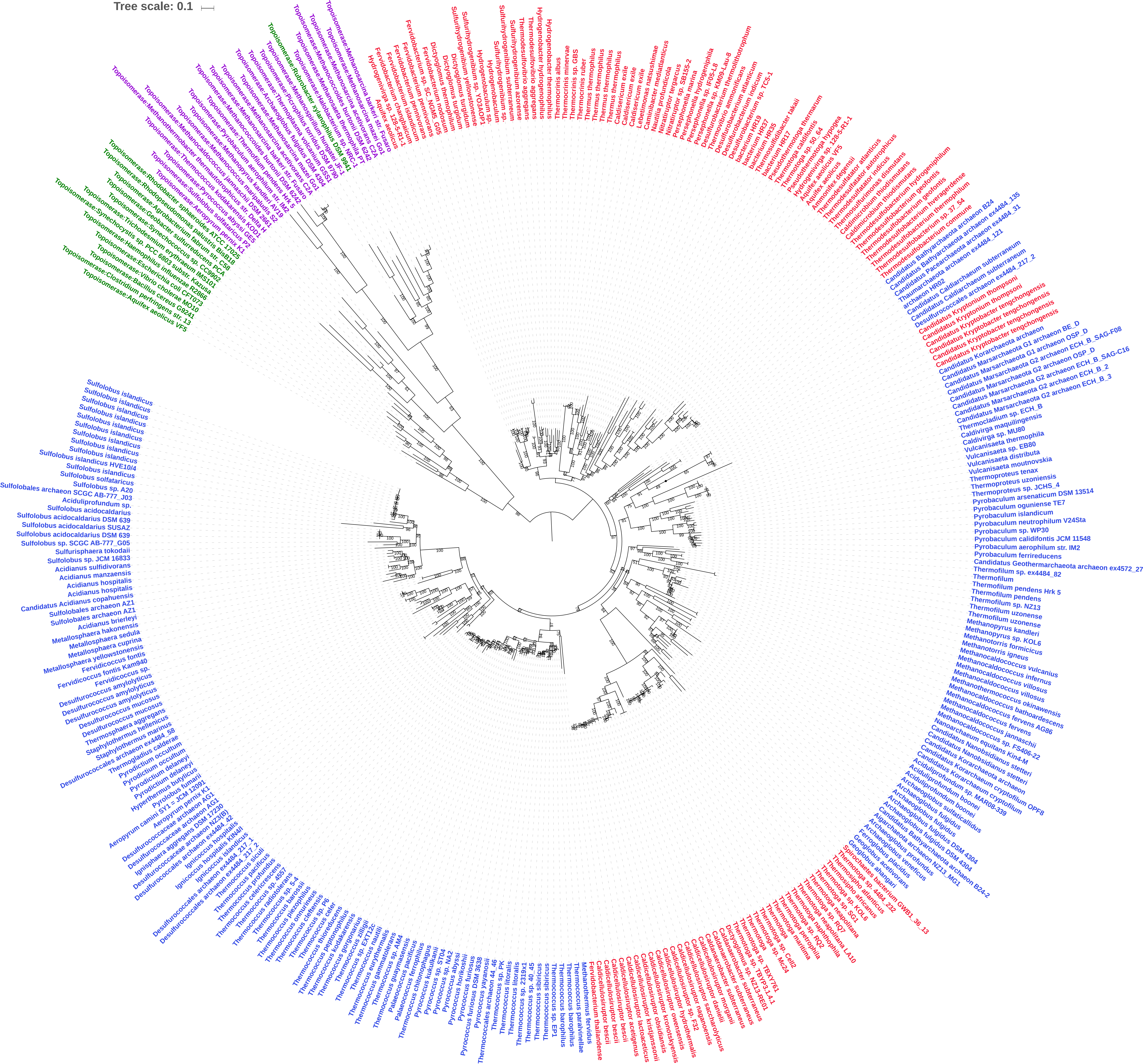
Tree scale: 0.1
Topoisomerase:Aquifex aeolicus VF5
Topoisomerase:Clostridium perfringens str. 13
Topoisomerase:Bacillus cereus G9241
Topoisomerase:Vibrio cholerae MO10
Topoisomerase:Escherichia coli CFT073
Topoisomerase:Haemophilus influenzae R2866
Topoisomerase:Synechocystis sp. PCC 6803 substr. Kazusa
Topoisomerase:Trichodesmium erythraeum IMS101
Topoisomerase:Synechococcus sp. CC9902
Topoisomerase:Geobacter sulfurreducens PCA
Topoisomerase:Agrobacterium fabrum str. C58
Topoisomerase:Rhodopseudomonas palustris BisB18
Topoisomerase:Rhodobacter sphaeroides ATCC 17025
Topoisomerase:Aeropyrum pernix K1
Topoisomerase:Sulfolobus solfataricus P2
Topoisomerase:Pyrococcus abyssi GE5
Topoisomerase:Thermococcus kodakarensis KOD1
Topoisomerase:Methanothermobacter thermautotrophicus str. Delta H
Topoisomerase:Methanocaldococcus jannaschii DSM 2661
Topoisomerase:Methanopyrus kandleri AV19
Topoisomerase:Methanococcus maripaludis S2
Topoisomerase:Pyrobaculum aerophilum str. IM2
Topoisomerase:Thermofilum pendens Hrk 5
Topoisomerase:Methanococcoides burtonii DSM 6242
Topoisomerase:Methanosarcina acetivorans C2A
Topoisomerase:Methanosarcina mazei Go1
Topoisomerase:Methanosarcina barkeri str. Fusaro
Topoisomerase:Archaeoglobus fulgidus DSM 4304
Topoisomerase:Picrophilus torridus DSM 9790
Topoisomerase:Thermoplasma volcanium GSS1
Topoisomerase:Rubrobacter xylanophilus DSM 9941
Topoisomerase:Methanospirillum hungatei JF-1
Topoisomerase:Halobacterium sp. NRC-1
Topoisomerase:Methanosaeta thermophila PT
Aquifex aeolicus
Hydrogenivirga sp. 128-5-R1-1
Fervidobacterium islandicum
Fervidobacterium changbaicum
Fervidobacterium pennivorans
Fervidobacterium sp. SC_NGM5_G05
Fervidobacterium nodosum
Dictyoglomus thermophilum
Dictyoglomus turgidum
Sulfurihydrogenibium sp. YO3AOP1
Sulfurihydrogenibium yellowstonense
Hydrogenobaculum sp.
Hydrogenobaculum
Sulfurihydrogenibium sp.
Sulfurihydrogenibium azorense
Sulfurihydrogenibium subterraneum
Thermodesulfovibrio aggregans
Thermodesulfovibrio aggregans
Hydrogenobacter hydrogenophilus
Hydrogenobacter thermophilus
Thermocrinis albus
Thermocrinis minervae
Thermocrinis sp. GBS
Thermocrinis ruber
Thermus thermophilus
Thermus thermophilus
Thermus thermophilus
Thermus thermophilus
Caldisericum exile
Caldisericum exile
Lebetimonas natsushimae
Caminibacter mediatlanticus
Nautilia profundicola
Nitratiruptor tergarcus
Nitratiruptor sp. SB155-2
Persephonella marina
Persephonella hydrogeniphila
Persephonella sp. IF05-L8
Persephonella sp. KM09-Lau-8
Thermovibrio ammonificans
Desulfurobacterium thermolithotrophum
Desulfurobacterium atlanticum
Desulfurobacterium indicum
Desulfurobacterium sp. TC5-1
bacterium HR19
bacterium HR37
bacterium HR35
bacterium HR17
Thermosulfidibacter takaii
Pseudothermotoga thermarum
Thermotoga caldifontis
Thermotoga sp. 50_64
Pseudothermotoga hypogea
Hydrogenivirga sp. 128-5-R1-1
Aquifex aeolicus VF5
Aquifex aeolicus
Ammonifex degensii
Thermodesulfatator atlanticus
Thermodesulfatator autotrophicus
Thermodesulfatator indicus
Thermodesulfurimonas dismutans
Caldimicrobium thiodismutans
Thermodesulfobacterium hydrogeniphilum
Thermodesulfobacterium geofontis
Thermodesulfobacterium geofontis
Thermodesulfobacterium hveragerdense
Thermodesulfobacterium thermophilum
Thermodesulfobacterium sp. 37_54
Thermodesulfobacterium commune
Candidatus Bathyarchaeota archaeon B24
Candidatus Bathyarchaeota archaeon ex4484_135
Candidatus Pacearchaeota archaeon ex4484_31
Thaumarchaeota archaeon ex4484_121
Candidatus Caldiarchaeum subterraneum
archaeon HR02
Candidatus Caldiarchaeum subterraneum
Desulfurococcales archaeon ex4484_217_2
Candidatus Kryptonium thompsoni
Candidatus Kryptonium thompsoni
Candidatus Kryptobacter tengchongensis
Candidatus Kryptobacter tengchongensis
Candidatus Kryptobacter tengchongensis
Candidatus Kryptobacter tengchongensis
Candidatus Korarchaeota archaeon
Candidatus Marsarchaeota G1 archaeon BE_D
Candidatus Marsarchaeota G1 archaeon OSP_D
Candidatus Marsarchaeota G2 archaeon ECH_B_SAG-F08
Candidatus Marsarchaeota G2 archaeon OSP_D
Candidatus Marsarchaeota G2 archaeon ECH_B_SAG-C16
Candidatus Marsarchaeota G2 archaeon ECH_B_2
Candidatus Marsarchaeota G2 archaeon ECH_B_3
Thermocladium sp. ECH_B
Caldivirga maquilingensis
Caldivirga sp. MU80
Vulcanisaeta thermophila
Vulcanisaeta sp. EB80
Vulcanisaeta distributa
Vulcanisaeta moutnovskia
Thermoproteus tenax
Thermoproteus uzoniensis
Thermoproteus sp. JCHS_4
Pyrobaculum arsenaticum DSM 13514
Pyrobaculum oguniense TE7
Pyrobaculum islandicum
Pyrobaculum neutrophilum V24Sta
Pyrobaculum sp. WP30
Pyrobaculum calidifontis JCM 11548
Pyrobaculum aerophilum str. IM2
Pyrobaculum ferrireducens
Candidatus Geothermarchaeota archaeon ex4572_27
Thermofilum sp. ex4484_82
Thermofilum
Thermofilum pendens Hrk 5
Thermofilum pendens
Thermofilum sp. NZ13
Thermofilum uzonense
Thermofilum uzonense
Methanopyrus kandleri
Methanopyrus sp. KOL6
Methanotorris formicicus
Methanotorris igneus
Methanocaldococcus vulcanius
Methanocaldococcus infernus
Methanocaldococcus villosus
Methanocaldococcus villosus
Methanothermococcus okinawensis
Methanocaldococcus bathoardescens
Methanocaldococcus fervens AG86
Methanocaldococcus fervens
Methanocaldococcus jannaschii
Methanocaldococcus sp. FS406-22
Nanoarchaeum equitans Kin4-M
Candidatus Nanobsidianus stetteri
Candidatus Nanobsidianus stetteri
Candidatus Korarchaeum cryptofilum OPF8
Candidatus Korarchaeota archaeon
Candidatus Korarchaeum cryptofilum
Aciduliprofundum sp. MAR08-339
Aciduliprofundum boonei
Aciduliprofundum boonei
Archaeoglobus sulfaticallidus
Archaeoglobus fulgidus
Archaeoglobus fulgidus DSM 4304
Archaeoglobus fulgidus DSM 4304
Candidatus Bathyarchaeota archaeon B24-2
Archaeoglobales archaeon NZ13_MG1
Archaeoglobus profundus
Archaeoglobus veneficus
Geoglobus acetivorans
Ferroglobus placidus
Geoglobus ahangari
Spirochaetes bacterium GWB1_36_13
Thermosipho africanus
Thermotoga sp. 4484_232
Thermosipho atlanticus
Thermotoga sp. KOL6
Thermotoga sp. SG1
Thermotoga neapolitana
Thermotoga neapolitana LA10
Thermotoga sp. RQ7
Thermotoga naphthophila
Thermotoga sp. RQ2
Thermotoga petrophila
Thermotoga maritima
Thermotoga sp. Cell2
Thermotoga sp. Mc24
Thermotoga sp. TBYP3.1.4.1
Thermotoga sp. TBXY761
Thermotoga sp. NZ13-RE01
Caldanaerobacter subterraneus
Caldanaerobacter subterraneus
Caldicellulosiruptor morganii
Caldicellulosiruptor saccharolyticus
Caldicellulosiruptor danielii
Caldicellulosiruptor naganoensis
Caldicellulosiruptor sp. F32
Caldicellulosiruptor hydrothermalis
Caldicellulosiruptor kronotskyensis
Caldicellulosiruptor owensensis
Caldicellulosiruptor obsidiansis
Caldicellulosiruptor kristjanssonii
Caldicellulosiruptor lactoaceticus
Caldicellulosiruptor acetigenus
Caldicellulosiruptor bescii
Caldicellulosiruptor bescii
Caldicellulosiruptor bescii
Fervidobacterium thailandense
Methanothermus fervidus
Thermococcus paralvinellae
Thermococcus barophilus
Thermococcus sp. EP1
Thermococcus sibiricus
Thermococcus sibiricus
Thermococcus sp. 40_45
Thermococcus sp. 2319x1
Thermococcus litoralis
Thermococcus litoralis
Thermococcus sp. PK
Thermococcales archaeon 44_46
Pyrococcus yayanosii
Pyrococcus furiosus DSM 3638
Pyrococcus furiosus
Pyrococcus horikoshii
Pyrococcus abyssi
Pyrococcus sp. NA2
Pyrococcus sp. ST04
Pyrococcus kukulkanii
Thermococcus chitonophagus
Palaeococcus ferrophilus
Palaeococcus pacificus
Thermococcus guaymasensis
Thermococcus sp. AM4
Thermococcus gammatolerans
Thermococcus eurythermalis
Thermococcus nautili
Thermococcus sp. EXT12c
Thermococcus zilligii
Thermococcus gorgonarius
Thermococcus kodakarensis
Thermococcus peptonophilus
Thermococcus thioreducens
Thermococcus celer
Thermococcus sp. P6
Thermococcus cleftensis
Thermococcus onnurineus
Thermococcus piezophilus
Thermococcus barossii
Thermococcus radiotolerans
Thermococcus sp. 5-4
Thermococcus sp. 4557
Thermococcus celericrescens
Thermococcus profundus
Thermococcus pacificus
Thermococcus siculi
Desulfurococcales archaeon ex4484_217_1
Ignicoccus islandicus
Ignicoccus hospitalis KIN4/I
Ignicoccus hospitalis
Desulfurococcales archaeon ex4484_42
Desulfurococcaceae archaeon NZ23(B)
Ignisphaera aggregans DSM 17230
Desulfurococcaceae archaeon AG1
Aeropyrum camini SY1 = JCM 12091
Aeropyrum pernix K1
Pyrolobus fumarii
Hyperthermus butylicus
Pyrodictium delaneyi
Pyrodictium delaneyi
Pyrodictium occultum
Pyrodictium occultum
Thermogladius calderae
Desulfurococcales archaeon ex4484_58
Staphylothermus marinus
Staphylothermus hellenicus
Thermosphaera aggregans
Desulfurococcus mucosus
Desulfurococcus mucosus
Desulfurococcus amylolyticus
Desulfurococcus amylolyticus
Desulfurococcus amylolyticus
Fervidicoccus sp.
Fervidicoccus fontis Kam940
Fervidicoccus fontis
Metallosphaera yellowstonensis
Metallosphaera cuprina
Metallosphaera sedula
Metallosphaera hakonensis
Acidianus brierleyi
Sulfolobales archaeon AZ1
Sulfolobales archaeon AZ1
Candidatus Acidianus copahuensis
Acidianus hospitalis
Acidianus hospitalis
Acidianus manzaensis
Acidianus sulfidivorans
Sulfolobus sp. JCM 16833
Sulfurisphaera tokodaii
Sulfolobus sp. SCGC AB-777_G05
Sulfolobus acidocaldarius DSM 639
Sulfolobus acidocaldarius SUSAZ
Sulfolobus acidocaldarius DSM 639
Sulfolobus acidocaldarius
Aciduliprofundum sp.
Sulfolobales archaeon SCGC AB-777_J03
Sulfolobus sp. A20
Sulfolobus solfataricus
Sulfolobus islandicus
Sulfolobus islandicus HVE10/4
Sulfolobus islandicus

Supplementary Figure 13. Phylogenetic reconstruction of RG dataset with TopR2-like paralogues removed. Bacterial and Archaeal topoisomerase sequences included as an outgroup to root the tree. Branch labels show species source of RG sequence, with Archaea in blue and Bacteria in red; or topoisomerase source, with Archaea in purple, Bacteria in green. Ultrafast bootstrap values indicated on branches as percentages.

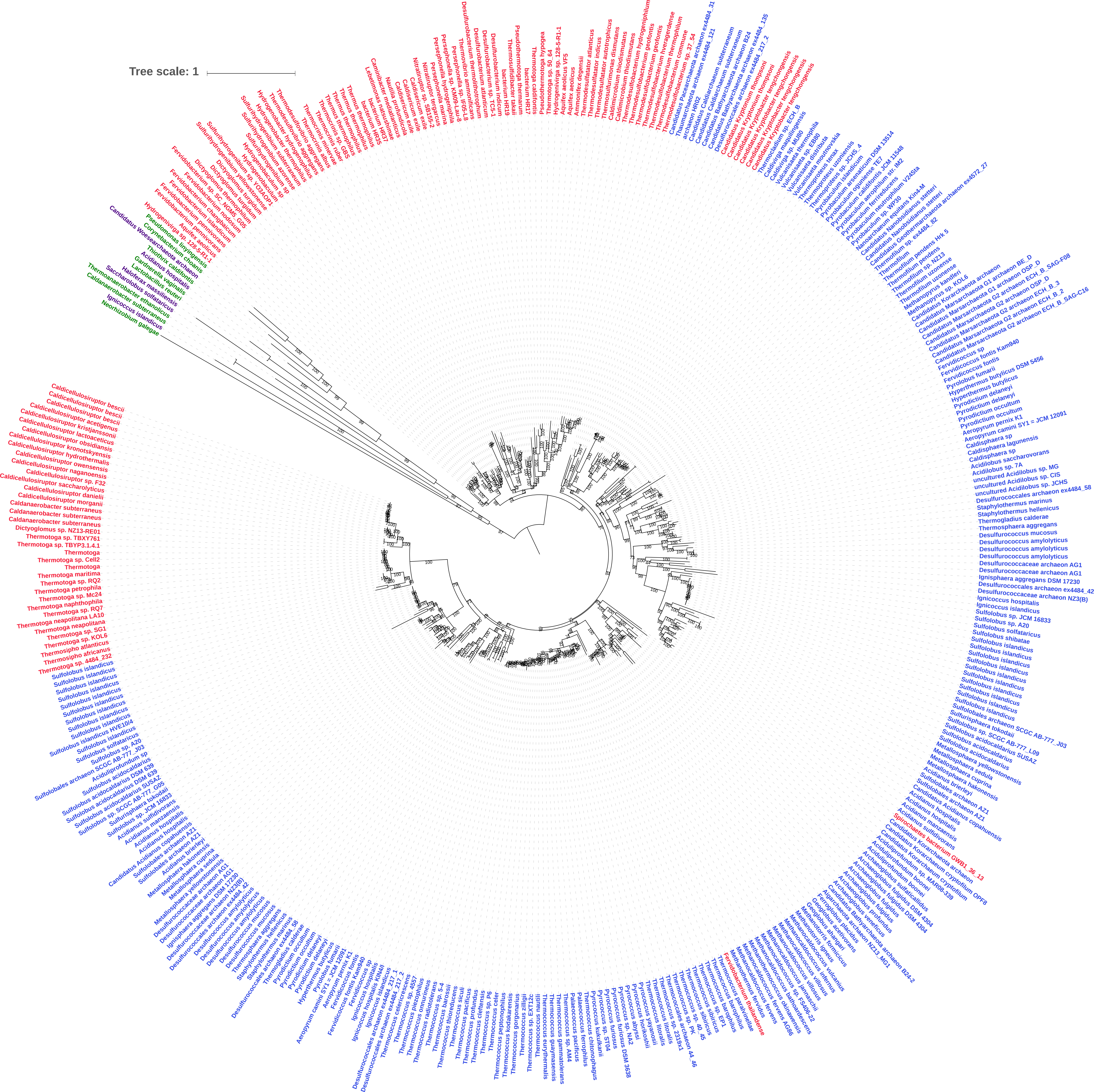
Tree scale: 1
Caldicellulosiruptor bescii
Caldicellulosiruptor bescii
Caldicellulosiruptor bescii
Caldicellulosiruptor acetigenus
Caldicellulosiruptor kristjanssonii
Caldicellulosiruptor lactoaceticus
Caldicellulosiruptor obsidiansis
Caldicellulosiruptor kronotskyensis
Caldicellulosiruptor hydrothermalis
Caldicellulosiruptor owensensis
Caldicellulosiruptor naganoensis
Caldicellulosiruptor sp. F32
Caldicellulosiruptor saccharolyticus
Caldicellulosiruptor danielii
Caldicellulosiruptor morganii
Caldanaerobacter subterraneus
Caldanaerobacter subterraneus
Caldanaerobacter subterraneus
Dictyoglomus sp. NZ13-RE01
Thermotoga sp. TBXY761
Thermotoga sp. TBYP3.1.4.1
Thermotoga
Thermotoga sp. Cell2
Thermotoga
Thermotoga maritima
Thermotoga sp. RQ2
Thermotoga petrophila
Thermotoga sp. Mc24
Thermotoga naphthophila
Thermotoga sp. RQ7
Thermotoga neapolitana LA10
Thermotoga neapolitana
Thermotoga sp. SG1
Thermotoga sp. KOL6
Thermosipho atlanticus
Thermosipho africanus
Thermotoga sp. 4484_232
Aeropyrum pernix K1
Aeropyrum camini SY1 = JCM 12091
Caldisphaera sp
Caldisphaera lagunensis
Caldisphaera sp
Acidilobus saccharovorans
Acidilobus sp. 7A
uncultured Acidilobus sp. MG
uncultured Acidilobus sp. CIS
uncultured Acidilobus sp. JCHS
Desulfurococcales archaeon ex4484_58
Staphylothermus marinus
Staphylothermus hellenicus
Thermogladius calderae
Thermosphaera aggregans
Desulfurococcus mucosus
Desulfurococcus amylolyticus
Desulfurococcus amylolyticus
Desulfurococcus amylolyticus
Desulfurococcaceae archaeon AG1
Desulfurococcaceae archaeon AG1
Ignisphaera aggregans DSM 17230
Desulfurococcales archaeon ex4484_42
Desulfurococcaceae archaeon NZ3(B)
Ignicoccus hospitalis
Ignicoccus islandicus
Sulfolobus sp. JCM 16833
Sulfolobus sp. A20
Sulfolobus solfataricus
Sulfolobus shibatae

Supplementary Figure 14. Phylogenetic reconstruction of RG dataset. Bacterial and Archaeal helicase sequences included as an outgroup to root the tree. Branch labels show species source of RG sequence, with Archaea in blue and Bacteria in red; or helicase source, with Archaea in purple, Bacteria in green. Ultrafast bootstrap values indicated on branches as percentages.

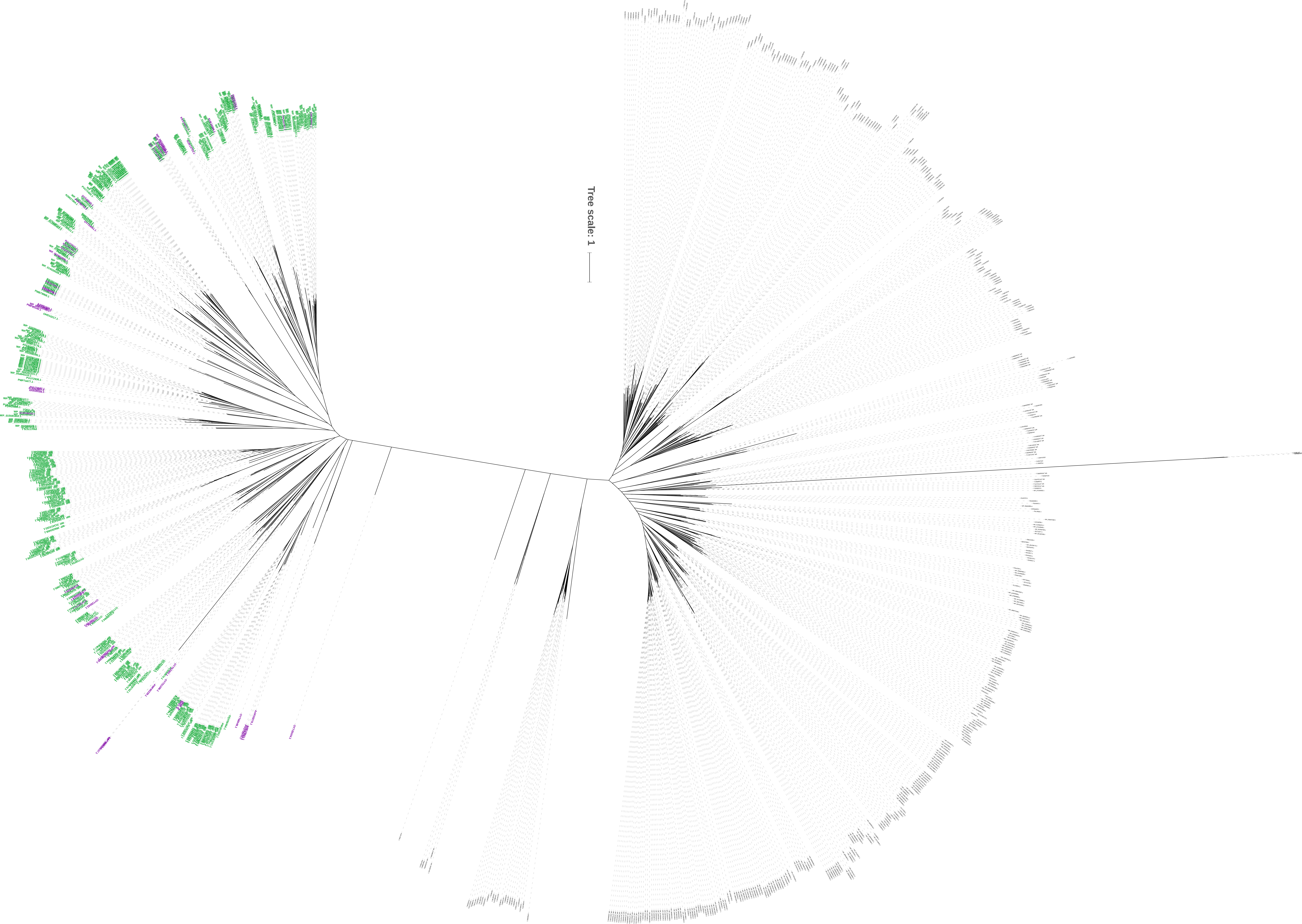
Tree scale: 1

Supplementary Figure 15. Phylogenetic tree generated from full HMMer hit results. Sequences identified as likely RG proteins by alignment with Swissprot sequences are labelled in green; sequences clustering with RG, but excluded by the alignment step are coloured in purple. Non-RG sequences (topoisomerase and helicase) sequences are labelled in black.

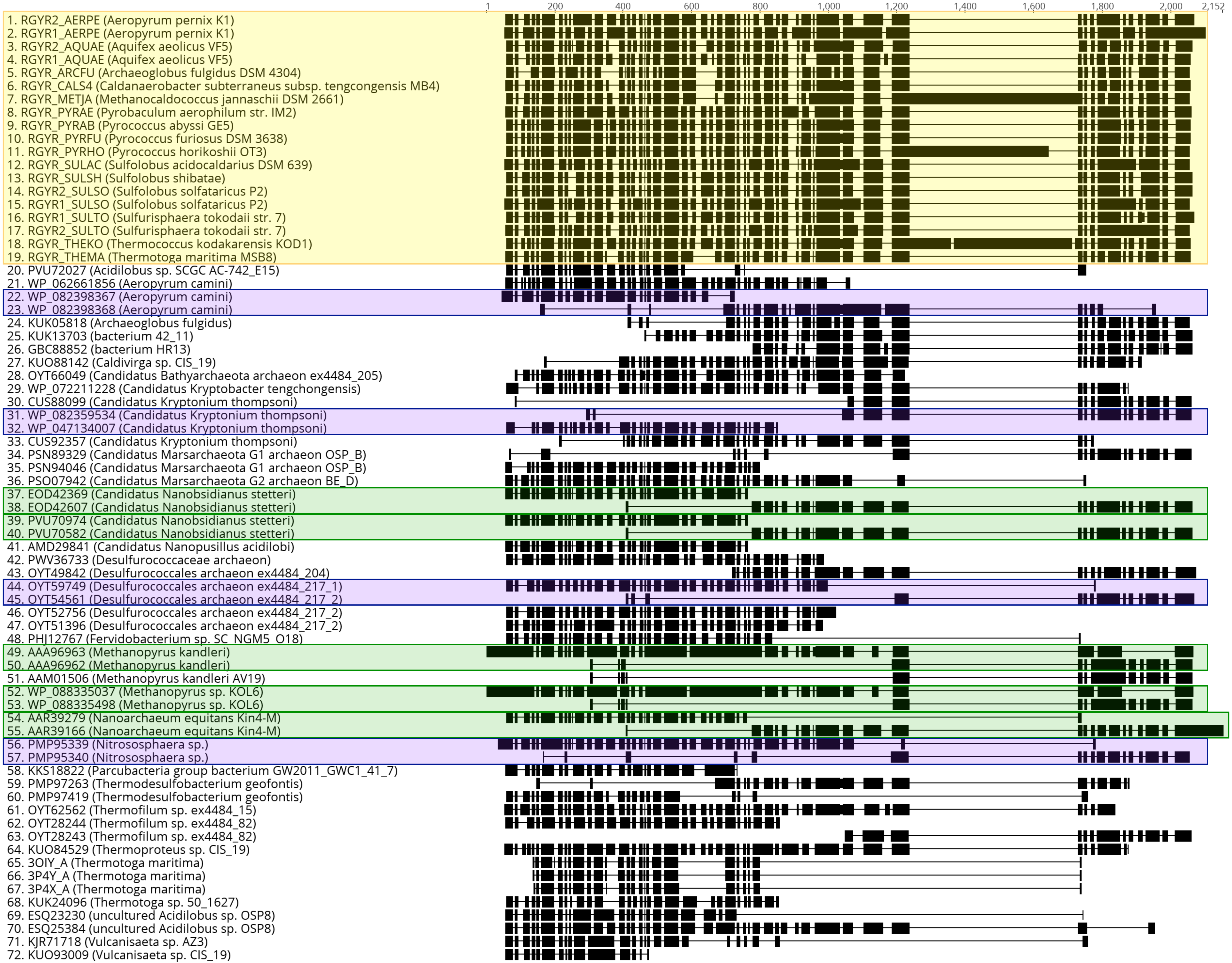
1
200
400
600
800
1,000
1,200
1,400
1,600
1,800
2,000
2,152
1. RGYR2_AERPE (Aeropyrum pernix K1)
2. RGYR1_AERPE (Aeropyrum pernix K1)
3. RGYR2_AQUAE (Aquifex aeolicus VF5)
4. RGYR1_AQUAE (Aquifex aeolicus VF5)
5. RGYR_ARCFU (Archaeoglobus fulgidus DSM 4304)
6. RGYR_CALS4 (Caldanaerobacter subterraneus subsp. tengcongensis MB4)
7. RGYR_METJA (Methanocaldococcus jannaschii DSM 2661)
8. RGYR_PYRAE (Pyrobaculum aerophilum str. IM2)
9. RGYR_PYRAB (Pyrococcus abyssi GE5)
10. RGYR_PYRFU (Pyrococcus furiosus DSM 3638)
11. RGYR_PYRHO (Pyrococcus horikoshii OT3)
12. RGYR_SULAC (Sulfolobus acidocaldarius DSM 639)
13. RGYR_SULSH (Sulfolobus shibatae)
14. RGYR2_SULSO (Sulfolobus solfataricus P2)
15. RGYR1_SULSO (Sulfolobus solfataricus P2)
16. RGYR1_SULTO (Sulfurisphaera tokodaii str. 7)
17. RGYR2_SULTO (Sulfurisphaera tokodaii str. 7)
18. RGYR_THEKO (Thermococcus kodakarensis KOD1)
19. RGYR_THEMA (Thermotoga maritima MSB8)
20. PVU72027 (Acidilobus sp. SCGC AC-742_E15)
21. WP_062661856 (Aeropyrum camini)
22. WP_082398367 (Aeropyrum camini)
23. WP_082398368 (Aeropyrum camini)
24. KUK05818 (Archaeoglobus fulgidus)
25. KUK13703 (bacterium 42_11)
26. GBC88852 (bacterium HR13)
27. KUO88142 (Caldivirga sp. CIS_19)
28. OYT66049 (Candidatus Bathyarchaeota archaeon ex4484_205)
29. WP_072211228 (Candidatus Kryptobacter tengchongensis)
30. CUS88099 (Candidatus Kryptonium thompsoni)
31. WP_082359534 (Candidatus Kryptonium thompsoni)
32. WP_047134007 (Candidatus Kryptonium thompsoni)
33. CUS92357 (Candidatus Kryptonium thompsoni)
34. PSN89329 (Candidatus Marsarchaeota G1 archaeon OSP_B)
35. PSN94046 (Candidatus Marsarchaeota G1 archaeon OSP_B)
36. PSO07942 (Candidatus Marsarchaeota G2 archaeon BE_D)
37. EOD42369 (Candidatus Nanobsidianus stetteri)
38. EOD42607 (Candidatus Nanobsidianus stetteri)
39. PVU70974 (Candidatus Nanobsidianus stetteri)
40. PVU70582 (Candidatus Nanobsidianus stetteri)
41. AMD29841 (Candidatus Nanopusillus acidilobi)
42. PWV36733 (Desulfurococcaceae archaeon)
43. OYT49842 (Desulfurococcales archaeon ex4484_204)
44. OYT59749 (Desulfurococcales archaeon ex4484_217_1)
45. OYT54561 (Desulfurococcales archaeon ex4484_217_2)
46. OYT52756 (Desulfurococcales archaeon ex4484_217_2)
47. OYT51396 (Desulfurococcales archaeon ex4484_217_2)
48. PHJ12767 (Fervidobacterium sp. SC_NGM5_O18)
49. AAA96963 (Methanopyrus kandleri)
50. AAA96962 (Methanopyrus kandleri)
51. AAM01506 (Methanopyrus kandleri AV19)
52. WP_088335037 (Methanopyrus sp. KOL6)
53. WP_088335498 (Methanopyrus sp. KOL6)
54. AAR39279 (Nanoarchaeum equitans Kin4-M)
55. AAR39166 (Nanoarchaeum equitans Kin4-M)
56. PMP95339 (Nitrososphaera sp.)
57. PMP95340 (Nitrososphaera sp.)
58. KKS18822 (Parcubacteria group bacterium GW2011_GWC1_41_7)
59. PMP97263 (Thermodesulfobacterium geofontis)
60. PMP97419 (Thermodesulfobacterium geofontis)
61. OYT62562 (Thermofilum sp. ex4484_15)
62. OYT28244 (Thermofilum sp. ex4484_82)
63. OYT28243 (Thermofilum sp. ex4484_82)
64. KUO84529 (Thermoproteus sp. CIS_19)
65. 3OIY_A (Thermotoga maritima)
66. 3P4Y_A (Thermotoga maritima)
67. 3P4X_A (Thermotoga maritima)
68. KUK24096 (Thermotoga sp. 50_1627)
69. ESQ23230 (uncultured Acidilobus sp. OSP8)
70. ESQ25384 (uncultured Acidilobus sp. OSP8)
71. KJR71718 (Vulcanisaeta sp. AZ3)
72. KUO93009 (Vulcanisaeta sp. CIS_19)

Supplementary Figure 16. Schematic of alignment of potential RG sequences identified during phylogenetic analyses which were excluded during alignment analyses of HMMer hits (see materials and methods). Protein sequence annotations and their respective species are indicated on the left, a schematic of sequence alignment shown on the right. Black boxes indicate regions with aligned residues, horizontal lines indicate gaps in alignment. Swissprot sequences are highlighted in yellow for reference of *bona fide* RG sequences. Sequences highlighted in green represent known, or anticipated split RG sequences (Nanoarchaea and *Methanopyrus* species). Sequences highlighted in purple indicate potential new split RG sequences. Non-highlighted sequences are likely truncated, or misannotated RG sequences.

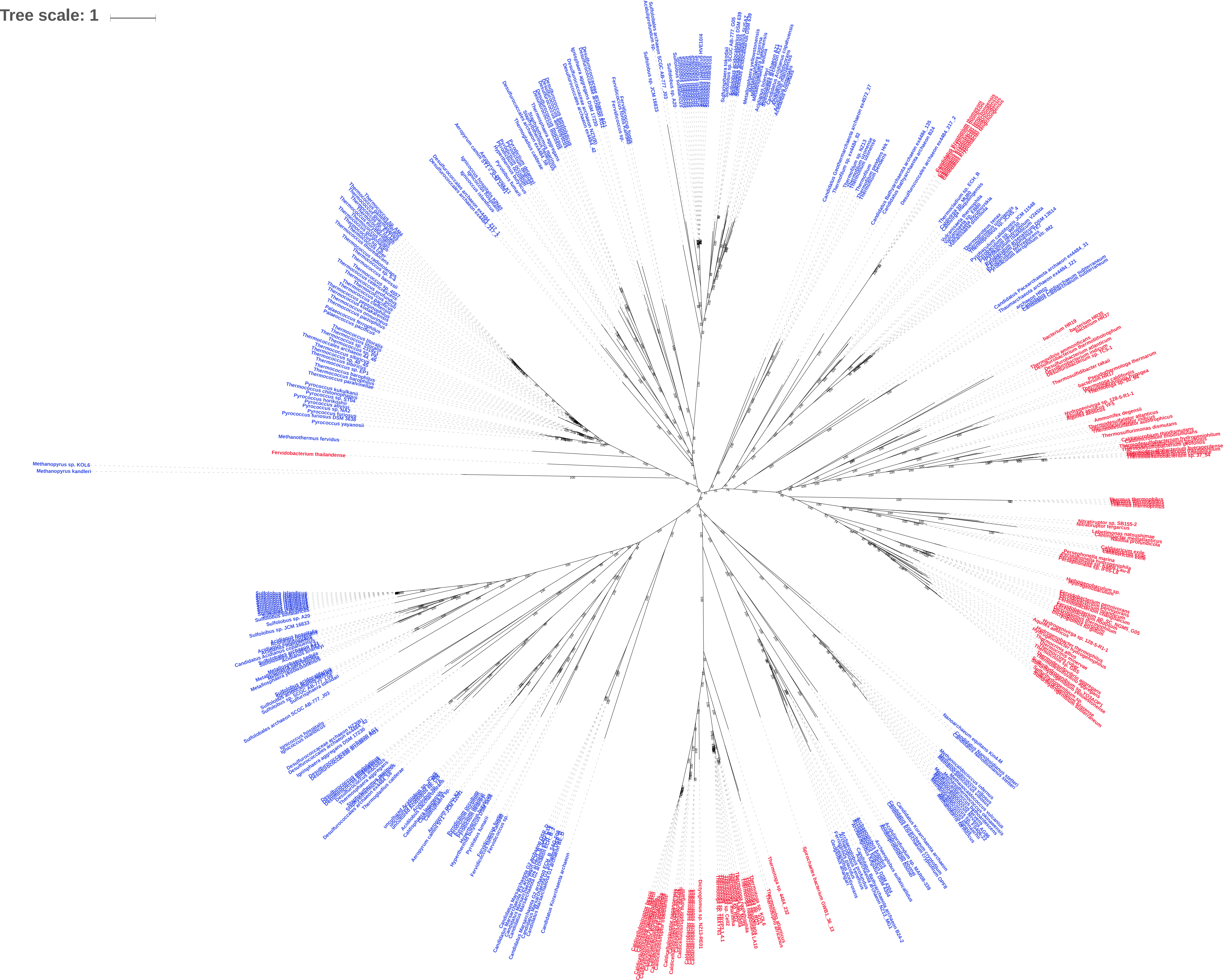
Tree scale: 1
Methanopyrus sp. KOL6
Methanopyrus kandleri
Fervidobacterium thailandense
Methanothermus fervidus
Pyrococcus yayanosii
Thermus thermophilus

Supplementary Figure 17. Phylogenetic reconstruction of entire RG dataset using minimal trimming of sequence alignment. Trimming was performed by Noisy, removing only 17% of positions. Branch labels show species source of RG sequence, with Archaea in blue and Bacteria in red.

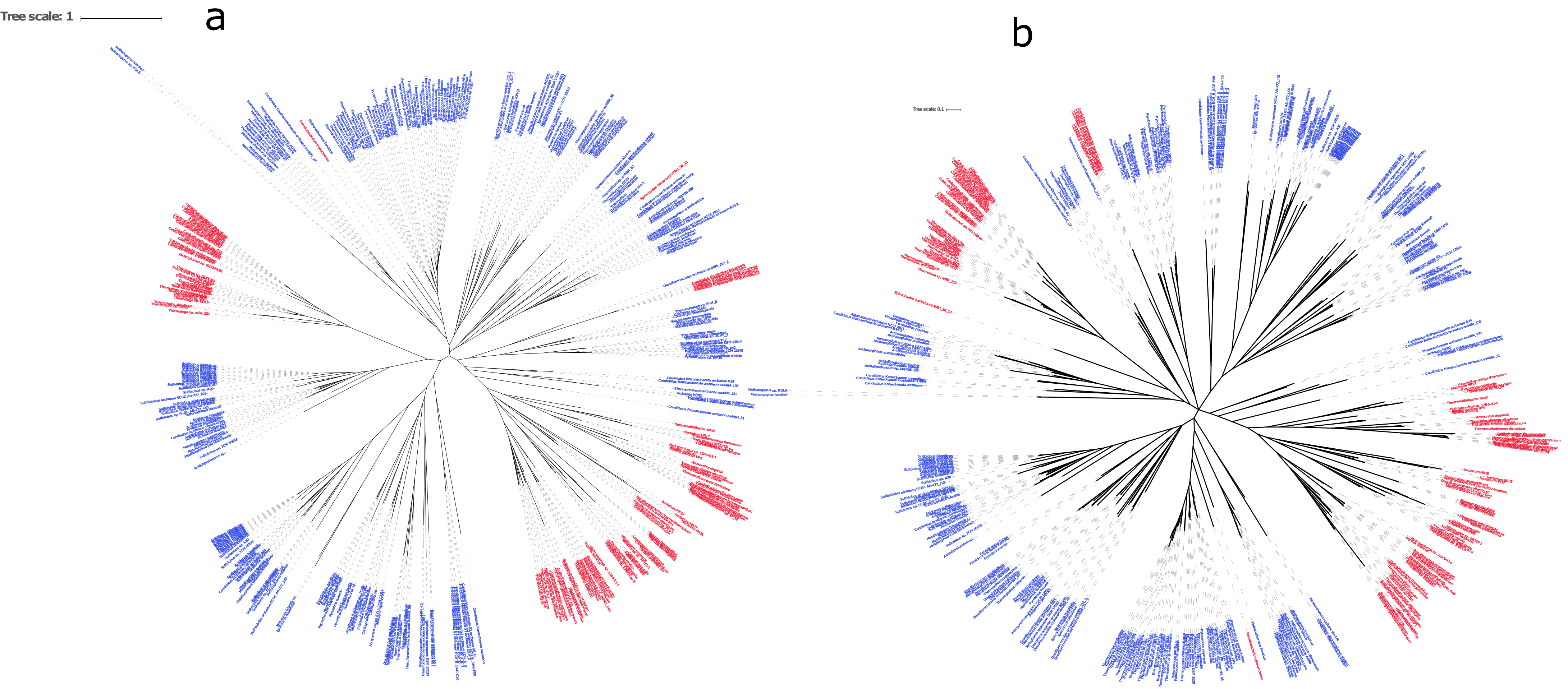
Tree scale: 1
a
b
Tree scale: 0.1

Supplementary Figure 18. Phylogenetic reconstructions of entire RG dataset using a) RaxML and b) MrBayes in place of IQ-TREE. None of these tree reconstruction methods recovers the monophyly of bacterial and archaeal RG.
